# Prognosis of lasso-like penalized Cox models with tumor profiling improves prediction over clinical data alone and benefits from bi-dimensional pre-screening

**DOI:** 10.1186/s12885-022-10117-1

**Published:** 2022-10-05

**Authors:** Rémy Jardillier, Dzenis Koca, Florent Chatelain, Laurent Guyon

**Affiliations:** 1 IRIG, Biosanté U1292, Univ. Grenoble Alpes, Inserm, CEA, Grenoble, France; 2 GIPSA-lab, Institute of Engineering University Grenoble Alpes, Univ. Grenoble Alpes, CNRS, Grenoble INP, Grenoble, France

**Keywords:** Cox model, Prediction, Survival model, Penalized regression, Lasso, RNA-seq, Cancer

## Abstract

**Background:**

Prediction of patient survival from tumor molecular ‘-omics’ data is a key step toward personalized medicine. Cox models performed on RNA profiling datasets are popular for clinical outcome predictions. But these models are applied in the context of “high dimension”, as the number *p* of covariates (gene expressions) greatly exceeds the number *n* of patients and *e* of events. Thus, pre-screening together with penalization methods are widely used for dimensional reduction.

**Methods:**

In the present paper, (i) we benchmark the performance of the lasso penalization and three variants (i.e., ridge, elastic net, adaptive elastic net) on 16 cancers from TCGA after pre-screening, (ii) we propose a bi-dimensional pre-screening procedure based on both gene variability and *p*-values from single variable Cox models to predict survival, and (iii) we compare our results with iterative sure independence screening (ISIS).

**Results:**

First, we show that integration of mRNA-seq data with clinical data improves predictions over clinical data alone. Second, our bi-dimensional pre-screening procedure can only improve, in moderation, the C-index and/or the integrated Brier score, while excluding irrelevant genes for prediction. We demonstrate that the different penalization methods reached comparable prediction performances, with slight differences among datasets. Finally, we provide advice in the case of multi-omics data integration.

**Conclusions:**

Tumor profiles convey more prognostic information than clinical variables such as stage for many cancer subtypes. Lasso and Ridge penalizations perform similarly than Elastic Net penalizations for Cox models in high-dimension. Pre-screening of the top 200 genes in term of single variable Cox model *p*-values is a practical way to reduce dimension, which may be particularly useful when integrating multi-omics.

**Supplementary Information:**

The online version contains supplementary material available at 10.1186/s12885-022-10117-1.

## Background

The roots of the ‘P4’ model of cancer medicine are based on prediction combined with personalization, prevention, and participation [1]. Prediction of the best treatment for a given patient and prediction of clinical outcome, including overall survival, are both of growing interest. The ‘-omics’ technologies now come with decreasing costs, which has made possible the molecular characterization of tumor samples of various subtypes, including gene expression [2, 3]. As a result, there are growing numbers of knowledge databases that include molecular profiling of patient tumors, together with clinical information from patient follow-up. Survival analysis from transcriptome profiling of cancer patients in terms of messenger RNA (mRNA) expression is now emerging for clinical use [4]. Transcriptome based tests such as MammaPrint ^®^ and Oncotype DX ^®^ are already used in clinical environment to assess risk of relapse of breast cancer, as well as Afirma ^®^ gene expression classifier that is used to differentiate between invasive cancer and benign nodules [5]. Also, [6–8] show that gene expression often provides the best survival prognosis compared with other omics.

The Cox proportional hazard model [9] is one of the most popular approaches in medicine to link covariates to survival data. When considering the number of covariates, *p* (which can typically be 20,000 gene products), in relation to the number of patients in the databases, *n*, and so the number of events *e* (which can typically be only a few hundred), various issues occur due to the high dimensionality [10], which include the lack of stability of the selected genes [11] and over-fitting [12]. This $p \gg e$ problem is referred to as the ‘curse of dimensionality’. The issues are aggravated when integrating multi-omics data [13], which is a research area of growing interest [14, 15]. Among many, there are two main distinct strategies to tackle issues arising from high dimensionality, both of which aim to reduce the number of variables considered: screening procedures and penalization methods [10, 16].

Cox regression model with the lasso penalty for variable selection [17] is often used to identify few prognostic biomarkers from among thousands of genes profiled, and to obtain a parsimonious model for simpler and cheaper clinical applications. Lasso generalizations have been proposed for generalized linear models, such as Cox regression, to improve the performance and stability. In particular, the elastic net [18] and the adaptive elastic net [19] are regularization procedures that can overcome some stability issues of the lasso in the presence of highly correlated variables [20], and the ridge penalty allows control of the variance of the estimator. The lasso tends to select one variable in a set of correlated predictors [21]. The elastic net and the adaptive elastic net result in lower mean-squared errors than the lasso and the ridge in the presence of highly correlated variables [22]. Although there is no selection, the ridge regression has shown promise and reliability for survival prediction using high-dimensional microarray data [23].

First, Bovelstad et al. [24] compared the ridge and the lasso among other prediction models on three real datasets only. Their results showed that the ridge penalty obtains the best predictions on the selected datasets. Then, Benner et al. [25] compared the ridge, lasso, elastic net, and adaptive lasso penalties on simulated and two real cancer datasets. As practical conclusions, the authors advocated for the use of lasso or elastic net penalization, as they do not require an initial estimation step, and were among the best-performing methods in their simulations. They also suggest further research on gene pre-screening prior to the use of Cox model. Finally, [26] showed that the ridge, lasso and elastic net penalties perform equally well for low-dimensional settings with few events.

Pre-screening methods should be considered as a statistical screening procedure to remove irrelevant genes. From this point of view, the aim is not to identify the most relevant variables, but to select a moderate size subset of variables that can be further reduced using penalized approaches [24, 27, 28]. Among many, two strategies are commonly used to pre-screen genes. The first is an unsupervised technique that aims at pre-screening the least variable genes among the patients. Indeed, the least variable genes are subjected to measurement noise and can provide poor contributions to distinguish between patients [27]. On the contrary, the most variable genes present a better signal-to-noise ratio and are easier to measure in practice for both research and clinical applications. Moreover, in the context of differentially expressed genes, pre-screening the least variable genes has been shown to increase the identification power in high dimension analyses with microarray data [29, 30]. The second methodology is supervised, as the survival data are used in a single variable Cox model for each gene. Both of these two supervised and unsupervised pre-screening techniques are single variable (i.e., the scores are calculated gene by gene), but more complex (and computationally intensive) techniques exist [16]. Thus, these two pre-screening methodologies represent simple and useful techniques to reduce the dimension before multivariable analysis. However, both selecting the most variable genes, and the genes which have the highest correlation with survival, require a threshold. But the pre-screening step is often carried out without justification of these thresholds for both unsupervised [31–33] and supervised [34, 35] techniques, and it has been shown that these values can have an impact on the selection process [30].

To the best of our knowledge, no independent benchmark of multivariable Cox regression (i.e, ridge, lasso, elastic net, adaptive elastic net) has been defined using mRNA-seq datasets of a large set of cancers of The Cancer Genome Atlas (TCGA, https://www.cancer.gov/tcga) with supervised and unsupervised pre-screening of the genes, and according to well-established evaluation metrics for prediction. In this context, the goals of this paper are to: (i) study the impact of pre-screening the genes based on their interquartile range (IQR) and *p*-value from a single variable Cox model on prediction accuracy, and propose a rationale toward setting a threshold; (ii) compare four multivariable Cox penalty methods (i.e., ridge, lasso, elastic net, adaptive elastic net) after a pre-screening step; (iii) compare the pre-screening methods we propose to a well-known algorithm, the iterative sure independence screening (ISIS) [28].

To evaluate the Cox regression methods for prediction of overall survival, we chose a panel of sixteen cancers from TCGA. The scripts used to reproduce the figures presented throughout the article are available in a github repository. We used R version 3.6 and 4.0.3 [36], the survival package [37], and ggplot2 package [38] to produce the figures.

## Methods

### Penalization methods for a sparse model

We consider four alternative penalties: the ridge regression [23], the lasso [17], the elastic net [18], and the adaptive elastic net [19]. Briefly, these methods consist of the addition of a penalty term to the log-pseudo-likelihood $l(\beta )$ before the maximization:

- The lasso
  $$\begin{aligned} \hat{\beta }(\mathrm {lasso}) = \underset{\beta }{\text {argmax }} l(\beta ) - \lambda || \beta ||_1 \end{aligned}$$
- The elastic net
  $$\begin{aligned} \hat{\beta }(EN) = \underset{\beta }{\text {argmax }} l(\beta ) - \lambda (\alpha || \beta ||_1 + \frac{1-\alpha }{2}|| \beta ||_2^2) \end{aligned}$$
- The ridge
  $$\begin{aligned} \hat{\beta }(\mathrm {ridge}) = \underset{\beta }{\text {argmax }} l(\beta ) - \lambda || \beta ||_2^2 \end{aligned}$$
- The adaptive elastic net (a two-step procedure)
  1. estimates the vector $\hat{\beta ^0}$ by maximizing *l* with the ridge regression.
  2. weights the elastic net penalty with the coefficient $\beta _j^0, j \in \{1,...,p\}$ computed in step 1:
  $$\begin{aligned} \hat{\beta }(AEN) = \underset{\beta }{\text {argmax }} l(\beta ) - \lambda \sum\limits_{j=1}^p \hat{w}_j \left( \alpha |\beta _j| + \tfrac{1-\alpha }{2}|\beta _j|^2\right) , \end{aligned}$$
  with $$\hat{w}_j = 1 /|\hat{\beta _j^0}|, j \in \{1,...,p\}$$.

We used the package glmnet [39] to estimate Cox penalized models. We selected the parameter $\lambda$ by minimizing the deviance in the cross-validation process, on the training dataset. For more details on the Cox model and the parameters used for penalties, we refer the reader to Supplementary Materials and Supplementary Fig. S1.

We further compared the prognostic performance with non-convex methods, namely smoothly clipped absolute deviation (SCAD) and the mimimax concave penalty (MCP) [40]. These methods were proposed to avoid known bias of the lasso, and are defined as follows:

- SCAD
  $$\begin{aligned} \hat{\beta }(\mathrm {SCAD}) = \underset{\beta }{\text {argmax }} l(\beta )&- \lambda || \beta ||_1&\mathrm {, if } || \beta ||_1 \le \lambda \\&- \frac{2 \gamma \lambda || \beta ||_1 - || \beta ||_1^2 - \lambda ^2}{2(\gamma - 1)}&\mathrm {, if } \lambda < || \beta ||_1 \le \gamma \lambda \\&- \frac{(\gamma + 1) \lambda ^2}{2}&\mathrm {, if } || \beta ||_1 > \gamma \lambda \end{aligned}$$
- MCP
  $$\begin{aligned} \hat{\beta }(\mathrm {MCP}) = \underset{\beta }{\text {argmax }} l(\beta )&- \lambda || \beta ||_1 + \frac{\beta ^2}{2\gamma }&\mathrm {, if } || \beta ||_1 \le \gamma \lambda \\&- \frac{\gamma \lambda ^2}{2}&\mathrm {, if } || \beta ||_1 > \gamma \lambda \end{aligned}$$

$\gamma$ is a tuning parameter of the MCP/SCAD penalty. We used the default parameter (3 for MCP and 3.7 for SCAD). We used the package ncvreg [41] to compute the Cox models penalized by MCP or SCAD. Benner and colleagues show that SCAD depends on pre-selection procedures, and they advise lasso and elastic net as being the methods of choice [25]. We will thus show most of the results with lasso-like penalties.

### Cox model assumption

To test for the proportional hazard assumption, we learn one Cox model with all patients with the ridge penalization for each cancers, and we apply a Schoenfeld residual test followed by Benjamini-Hochberg correction for multiple testing (Table 1) [42]. Only THYM has a *p*-value below 0.05, showing a low probability to verify proportional hazards. We anyway compute all the predictions for THYM, as some authors consider that even if hazards are not proportional, survival prediction can be evaluated with a Cox model [43]. Nevertheless results from THYM should be interpreted with care.

**Table 1:** *P*-value, after Benjamini-Hochberg correction, of the Schoenfeld residual test, applied on the Cox model with ridge penalization. THYM, for which the *p*-value is low, is highlighted in bold

ACC	BLCA	BRCA	CESC	COAD	ESCA	GBM	HNSC	KIRC	KIRP
0.71	0.26	0.38	0.66	0.71	0.66	0.62	0.38	0.38	0.38
LAML	LGG	LIHC	LUAD	LUSC	MESO	OV	PAAD	PRAD	READ
0.38	0.26	0.38	0.73	0.41	0.41	0.42	0.73	0.73	0.38
STAD	TGCT	THCA	**THYM**	UCEC	UVM				
0.38	0.38	0.38	**0.011**	0.71	0.62				

### Prediction performance metrics

**Fig. 1:**
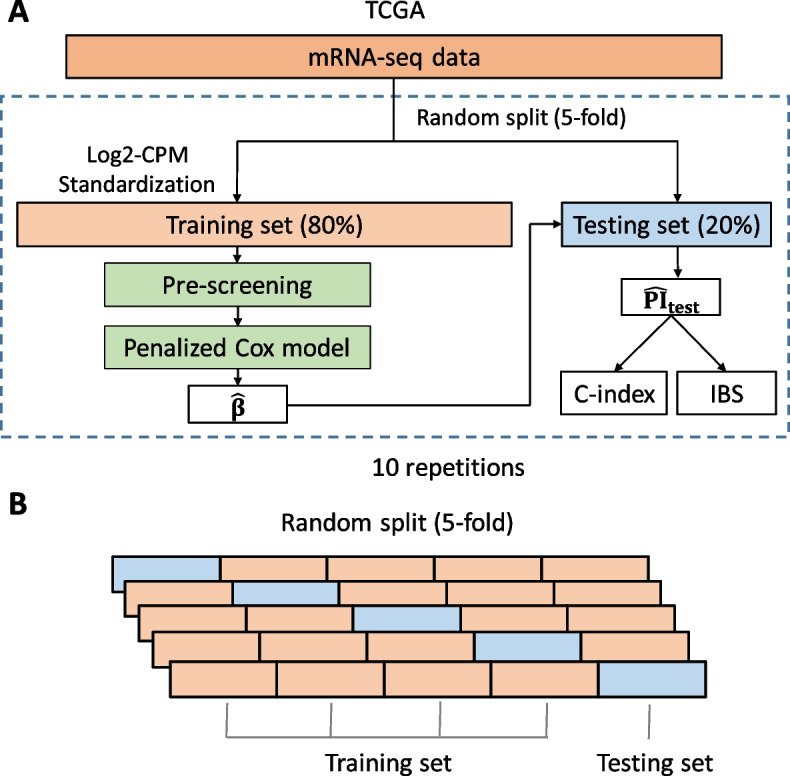
Procedure for the evaluation of prediction performances. CPM corresponds to Count Per Million normalization, PI means Prognostic Index and IBS refers to integrated Brier score

We estimate the prediction performance of the models by 10 repetitions of a K-fold cross-validation ($K=5$). We compute the $\beta$ vector of the Cox model on a training dataset ($\tfrac{4}{5}$ of the patients), and from this estimated vector we define a risk score for each patient in the testing dataset ($\tfrac{1}{5}$ of the patients). This risk score is called the ‘Prognostic Index’ (PI) and is defined for a given patient *i* as $\hat{\mathrm {PI}}_i = \hat{\beta }^T \mathbf {X}^i$, with $\hat{\beta }$ the estimator of the coefficients in the Cox model, and $\mathbf {X}$ the gene expression vector. This procedure allows assessment of prediction performance by computing the C-index and the Integrated Brier Score (IBS) (Fig. 1, [44]). Then, at the end of this classical procedure [15], 50 C-indices and 50 IBS are computed for each method. With this procedure, all patient measurements are used, either in the training set or in the testing set (but not both), and each measurement is used only once (no replacement).

The C-index allows the discrimination ability of a model to be assessed by quantifying the proportion of comparable patient pairs whose PI are in good agreement with their survival data. For two patients *i* and *j* with risk scores $\mathrm {PI}_i$ and $\mathrm {PI}_j$, and with survival times $T_i$ and $T_j$, the C-index is defined as $C = P(T_i < T_j \, | \, \text {PI}_i > \text {PI}_j)$. A C-index of 1 indicates perfect agreement, and a C-index of $\tfrac{1}{2}$ corresponds to random chance agreement. We took the estimator of the C-index given by [45] and theorized by [46]. In this estimator, only comparable pairs of patients are considered, meaning that the shortest time among both patients has to be measured (not censored).

The Brier Score [47] measures the average squared distance between the observed survival status and the predicted survival probability at a particular time *t*. It is always a number between 0 and 1, with 0 being the best possible value. We used the integrated Brier score that integrates the Brier Score between 0 and the maximum event time of the test set and divides this quantity by the maximum integration time. Then, while the C-index measures the ability of a model to rank patients according to their risks, the IBS estimates the capacity of a model to predict survival probabilities along time. The IBS is a global performance metric that assesses both discrimination and calibration, but is more difficult to interpret in practice. These two metrics are widely used to estimate prediction performance in practice and are complementary.

We used the package survcomp [48] to compute both the C-index and the IBS.

### The Cancer Genome Atlas dataset

Cancer acronyms, as provided by the TCGA consortium, are available in Supplementary Table S1. First, we included cancers available in TCGA for which there were more than 75 patients with mRNA-seq and survival data. Then, we followed recent formal recommendations [49] to exclude PCPG dataset that has too few events and SKCM dataset that has a high ratio of metastatic samples sequenced. We used overall survival as disease-outcome, except when the authors recommend the use of progression-free interval (BRCA, LGG, PRAD, READ, TGCT, THCA and THYM). After these two steps, we retained 26 cancers.

Finally, we computed the C-index estimates with the ridge regression method applied over all of the genes for these 26 cancers (Supplementary Fig. S2), as explained in Fig. 1, without the pre-screening step. To focus on cancers for which the prediction exceed a minimal level of performance with RNA-seq data, we decided to retain only the datasets for which the median C-index is significantly higher than 0.6 according to a one-sided Wilcoxon test at level 0.01. At the end of this procedure, we retained 16 cancer subtypes (Table 2). Among them, UVM, ACC, and KIRP contain few events ($e < 50$). Supplementary Fig. S3 shows that this may lead to a bias on the C-index toward a too optimistic value, resulting in an anti-correlation between the C-index and the number of events (cor = -0.63, p-val = 0.008). This anti-correlation disappears when selecting cancer subtypes for which the TCGA dataset contains more than 50 events (cor = -0.18, p-val = 0.55). However, we retain these 3 datasets as this bias is identical for all methods applied to a given dataset; therefore, the results remain comparable within a cancer subtype.

We obtained clinical and mRNA-seq datasets using the Broad GDAC FIREHOSE utility (https://gdac.broadinstitute.org). We retained only expressed genes (i.e. CPM value higher than 1) for at least 1% of the patients. Then, we used a log2-CPM normalization (count per million) of the mRNA-seq count data using packages edgeR [50] and limma [51], and we standardized the data in the training dataset, and in the testing datasets using the mean and standard deviation values of the training data.

**Table 2:** Characteristics of the 16 cancers selected. C-indices are computed with 10 repetitions of 5-fold cross-validation with the ridge regression and all of the genes in the Cox model. Datasets are ordered according to their median C-index (decreasing order)

Cancer	n events	n patients	Censoring rate	Survival - 3 years	C-index
ACC	27	77	0.65	0.75	0.86
KIRP	42	269	0.84	0.87	0.83
UVM	21	77	0.73	0.74	0.79
MESO	73	85	0.14	0.19	0.75
KIRC	175	526	0.67	0.76	0.73
LGG	192	510	0.62	0.56	0.73
CESC	70	288	0.76	0.72	0.7
PRAD	92	490	0.81	0.8	0.69
LIHC	125	358	0.65	0.62	0.67
UCEC	91	541	0.83	0.83	0.67
BLCA	165	371	0.56	0.48	0.65
LAML	92	149	0.38	0.31	0.65
BRCA	145	1079	0.87	0.88	0.64
HNSC	210	491	0.57	0.57	0.64
PAAD	93	175	0.47	0.34	0.63
LUAD	179	488	0.63	0.61	0.62

### The independent dataset

To further compare the validity of our prediction and the procedure used, we chose the clear cell renal cell carcinoma (ccRCC), for which the predictions are high (C-index = 0.75), and the dataset is large enough to gather a large number of events (*e* = 175). We collected the expression data and the clinical data for Renal Cell Carcinoma (RECA-EU) from the ICGC repositories (https://dcc.icgc.org), while selecting only ccRCC patients. We retained only expressed genes (i.e. CPM value higher than 1) for at least 1% of the patients. Then, we used a log2-CPM normalization (count per million) of the mRNA-seq count data using packages edgeR [50] and limma [51]. To test the procedure on an independent dataset, we used TCGA:KIRC as a training dataset while ICGC:RECA dataset as the testing dataset, in a similar way as explained above. Due to differences between datasets, we selected only genes that were found in both datasets. Therefore, we used 17,000 genes to learn the model on training subset of TCGA:KIRC and then tested both on the remaining patients from TCGA:KIRC or on all the patients in the independent ICGC:RECA dataset separately. We standardized the external testing dataset independently from the training set, by using the mean and standard deviation of the testing set. This standardizing procedure is different as for the K-fold validation, but was necessary to reach good performance in terms of IBS. We further comment this issue in the discussion section.

### Integration of mRNA-seq data together with clinical data

**Fig. 2:**
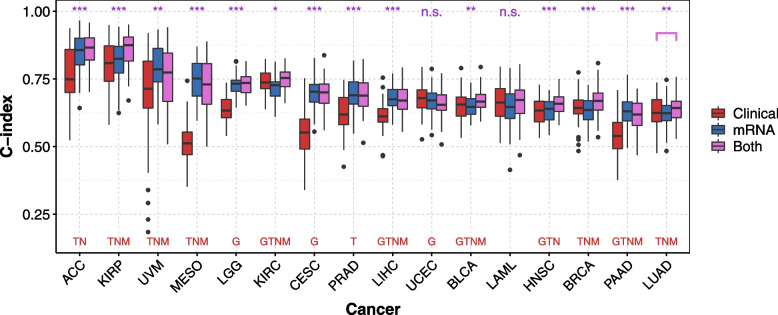
C-indices obtained with clinical data alone (red), mRNA-seq data alone with ridge penalty and all the genes (blue, no bi-dimensional pre-screening), and clinical and mRNA-seq data together (purple) for the 16 cancers studied. We computed the C-indices by 10 repetitions of a K-fold cross-validation (K=5). To test whether the added value of mRNA-seq data for prediction is significant over clinical data alone, we computed the *p*-values of a one-sided Wilcoxon signed-rank test (purple stars at the top of each graphic, Benjamini-Hochberg correction for the 16 *p*-values). Red letters at the bottom of each graphics indicate the clinical data available (G: grade; T: tumor; N: node; M: metastasis). Age is available for all cancers, and gender only for non-unisexual cancers (CESC, PRAD, TGCT are sex-specific). ***: $p \le 0.001$, **: $p \le 0.01$, *: $p \le 0.05$, +: $p \le 0.1$, n.s. : $p > 0.1$

Taking into account clinical information when assessing the predictive value of omics data is an important aspect of model building [52]. An added value of mRNA-seq data over clinical data alone to predict survival has been shown for some cancers but not all [7, 34, 44, 53]. Different strategies exist for combining mRNA-seq and clinical data [54, 55]. In this study, we added the prognostic indices computed with mRNA-seq data alone ($\mathrm {PI}_{mRNA}$) to classical clinical features (age, gender, grade, T, N, M), when available ($\mathrm {PI} = \beta \mathrm {PI}_{mRNA} + \sum _k \beta _k \mathrm {Clin}_k$, where $\mathrm {Clin}_k$ is the $k^{th}$ clinical variable). The TNM Staging System corresponds to a score constructed with the extent of the tumor (T), the extent of spread to the lymph nodes (N), and the presence of metastasis (M). We did not include gender for sex-specific cancers (CESC, PRAD, TGCT). Age is available for all cancers, and we specify whether the other variables are available in Fig. 2 and Supplementary Fig. S4 and S5. For example, the grade (G) is only available for 10 cancer subtypes out of 26.

We used the ridge penalty with all the genes to compute $\mathrm {PI}_{mRNA}$. To test if the mRNA-seq data added prediction quality over clinical data, we compared the C-index (resp. IBS) distributions obtained with clinical data alone and with both clinical and mRNA data by performing a one-sided Wilcoxon signed rank test for each of the 16 cancers studied.

### Bi-dimensional pre-screening procedure

#### Supervised pre-screening: based on the single variable Cox model

Single variable Cox pre-screening consists of allocating one *p*-value associated with the test ‘$\beta = 0$’ for each gene individually using the patients from the training dataset. We computed the *p*-values using the likelihood ratio test [56] implemented in the package survival [57]. We corrected for multiple testing with the Benjamini-Hochberg procedure [42]. We used six different thresholds (0.01, 0.05, 0.1, 0.2, 0.5, 1), and only the genes with a corrected *p*-value below a given threshold were kept to estimate $\beta _j$ in a multivariable Cox model.

This supervised pre-screening approach retains the genes that are individually associated with survival.

#### Unsupervised pre-screening: based on the interquartile range

We used the interquartile range (IQR) applied to the gene expression of patients from the training dataset for unsupervised pre-screening. The IQR is a robust measurement of the dispersion, and it is defined as the difference between the 75th and 25th percentiles.

The RNA-seq technology induces a relationship between the median and the IQR for expression data: the larger the median of the count data, the greater the IQR (Supplementary Fig. S6 A, B). The use of logarithm (base 2) in log2-CPM data allows to overcome the problem of asymmetric data distribution [58], but assigns a higher IQR to low count data because of the concave shape of the logarithm function (Supplementary Fig. S6 C).

To overcome this median-IQR trend bias, we used a variance stabilizing transformation (VST) algorithm [59]. The goal is to compensate for this tendency so that screening with the IQR does not penalize genes with low count data (CPM normalization) or high count data (log2-CPM normalization, Supplementary Fig. S6 D). Both of these class of genes can be important in the multivariable Cox model for prediction, and have to be treated equally in the screening process.

We excluded genes for which IQR of the VST data among all of the patients was below a given threshold (0, 0.5, 1, 1.5, 2, 2.5).

### Suggested screening algorithm

In this study, we suggest to screen the genes in a bi-dimensional way, both on individual correlation with survival (i.e. *p*-value of the single variable Cox model) and variability of the genes among patients (IQR of VST data). For each pair of thresholds, we compute 50 C-indices (resp. 50 IBS) as explained in Fig. 1. Optimal thresholds are those that maximize (resp. minimize) the median C-index (resp. IBS), according to the performance metric of interest (i.e. C-index or IBS in this study). The pair of thresholds that maximizes prediction accuracy is referred to as the ‘optimal case’, ‘optimal pre-screening’ or ‘optimal thresholds’.

Evaluation of prediction performances with the same cross-validation procedure and datasets used for model selection may lead to an optimistically biased evaluation. To overcome this issue and measure the extent to which our bi-dimensional screening procedure improves predictions, we used a nested cross-validation procedure [60]. Briefly, we simply re-ran the procedure with new sampling after having learned optimal thresholds, i.e. we learned new models on new training and testing datasets but with the optimal bi-dimensional screening threshold computed in the first run of 10 cross-validations (K=5). To evaluate if the prediction quality is improved by the pre-screening step, we compared the C-index (resp. IBS) distributions without screening (i.e. all the genes) and with the optimal pre-screening with boxplots. To help guide the comparisons, we also performed a one-sided Wilcoxon signed-rank tests, which is purely indicative as detailed in the discussion paragraph. We corrected the *p*-values with Benjamini-Hochberg method. To further compare prediction performances of the different form of penalizations, we did a one-sided Wilcoxon test at level 0.05 between distributions of the C-indices and IBS for each cancers.

### Simulation procedure

In order to evaluate the pre-screening procedure in a controlled environment, compatible with the Cox model, we ran simulations. The Cox model assumes the time dependent hazard, for a given patient *i*, to be $h^i(t) = h_0(t) . exp(\beta ^T \mathbf {X}^i)$. To compute the time dependent baseline $h_0(t)$, we used the Cox-Weibull model, $h_0(t) = r s t^{s-1}$ [61, 62]. The correlation structure of mRNA-seq data is complex and specific to each cancer [63]. Thus, we used the real data from TCGA for mRNA expression $\mathbf {X}^i$. We chose ccRCC as the cancer of interest for the same reasons as for the validation on an independent dataset: high C-index and large number of events. We learnt a Cox survival model with Elastic Net penalty 10 times, leading to sets of selected genes of size 51 to 75. Out of these sets, we selected the 68 genes that are selected at least 7 times among the 10 models. We then ran simulations only with this set of genes considered as the ground truth, and we use a normal distribution $\mathcal {N}(0, \sigma )$ to compute $\beta$. We ran the simulations for different values of $\sigma$ and chose $\sigma = 0.2$ as it leads to the closest Kaplan-Meier curves between simulated and real data (Supplementary Fig. S7). We simulate the survival time for a given patient *i* by

$$\begin{aligned} T^i = \left( \frac{-log(U) exp(-\beta ^T \mathbf {X}^i)}{r}\right) ^{1/s}, \end{aligned}$$

with (*r*, *s*) the parameters of the Cox-Weibull model, and *U* a variable following a standard uniform distribution [64]. Finally, we simulated censored time $C^i$ following a uniform distribution between 0 and $\theta$, this latter parameter determined to have the same censoring rate as for the real dataset (0.67 for the TCGA dataset considered) [65].

We ran only 3 different simulations scheme, due to the time required to run a single simulation, leading to 3 simulated datasets (each with 526 patients with the same gene expression as for the real datasets, but simulated survival and censored time). For each of the 3 datasets, we performed a pre-screening of the genes, and then learnt a Cox model with EN penalization, with the same K-fold with repetition procedure described above (Fig. 1). It leads to 50 measurements of C-index and IBS per pre-screening condition per simulation.

### Comparison with sure independence screening

Finally, we compared our bi-dimensional pre-screening procedure to the well-known sure independence screening [28]. This algorithm is close to the supervised screening procedure as it is based on individual correlation between each gene and the survival outcome. Briefly, a $|\beta |$ coefficient is computed individually for each gene in a single variable Cox model, and the *d* genes with the highest score are retained. The iterative procedure aims at handling possible spurious correlation and multicollinearity issues. The ISIS algorithm allows each gene that has not been selected at step *k* to enter the model at step $k+1$ based on their individual additional contribution in a multivariable Cox model with lasso penalty.

We followed the recommendations of [28] and set $d=\lfloor {\frac{n}{\log (n)}}\rfloor$. If we observe a convergence issue with this value, as has been mentioned by [55], we chose a lower value of *d* until the algorithm converge (i.e. $d=\lfloor {\frac{n}{2\log (n)}}\rfloor$ and then $d=\lfloor {\frac{n}{4\log (n)}}\rfloor$). We then apply the Cox model with ridge penalty on the genes selected by the ISIS procedure.

## Results

### mRNA-seq data improves predictions over clinical data alone for most of the investigated cancers

Adding mRNA-seq to clinical data improves predictions for 11 cancers according to the C-index, and for 5 cancers according to the IBS over the 16 cancers selected (Fig. 2, Supplementary Fig. S4). Overall, the predictions are significantly improved according to at least one of the metrics (i.e. C-index or IBS) for 13 cancers. These results show the benefit of mRNA data for survival prediction, and further encouraged us to compare the different penalization methods. For the 10 cancer subtypes not further considered for pre-screening, only Thymoma (THYM) showed an improvement. Nevertheless, the predictive power remains low (median C-index = 0.6), and we chose to keep only cancer subtypes for which we obtained adequate predictive power. In addition, the integration of clinical data can also improve predictions over mRNA data alone for 13 cancer subtypes out of 26 when evaluating with the C-index (7 out of the 16 cancers selected, and 6 out of the 10 cancers not further studied).

It is interesting to note that for MESO, although the stage, age, T, N and M are available, the predictions obtained from these clinical data are poor (median C-index of 0.51). On the other hand, for LAML, with only age and gender as clinical variables, we observe a median C-index of 0.66. Finally, for COAD and TGCT, the mRNA-seq data do not provide good predictions (median C-index $< 0.55$), whereas the clinical data does (median C-index $> 0.70$, Supplementary Fig. S5).

### Optimal bi-dimensional thresholds vary according to cancers, metrics, and penalty methods.

**Fig. 3:**
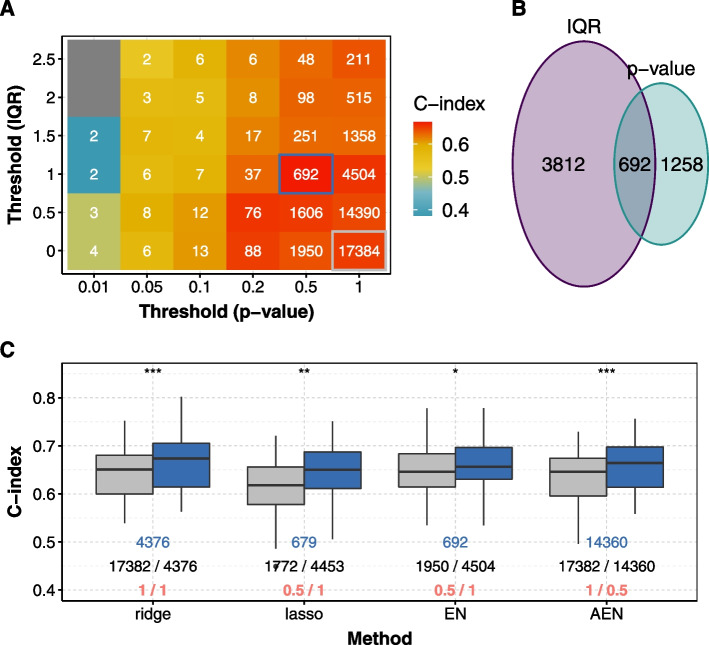
C-index computed with different pre-screening thresholds for BRCA and the elastic net penalty. (A) Median C-indices obtained by 10 repetitions of a K-fold cross-validation (K = 5) for different pre-screening thresholds for corrected *p*-values on the x-axis and for interquartile range (IQR) on the y-axis. Box surrounded by gray: no pre-screening; box surrounded by blue: optimal case (highest median C-index); white numbers: number of genes after the pre-screening step. (B) Number of genes retained by unsupervised (purple) and supervised (lightblue) pre-screening in the optimal case (the blue square in (A)). (C) Boxplot of C-indices obtained with 10 repetitions of 5-fold nested cross-validation without pre-screening (gray box in (A) for elastic net) and in the optimal case for bi-dimensional pre-screening (blue box in (A) for elastic net) for the ridge, the lasso, the elastic net (EN), the adaptive elastic net (AEN). The *p*-value above each method is calculated with a one-sided Wilcoxon signed-rank test between the C-indices obtained in the optimal case and without pre-screening; blue numbers are the number of genes retained after optimal bi-dimensional pre-screening; black and red numbers are respectively the number of genes and the optimal thresholds retained by supervised (left) and unsupervised (right) pre-screening in the optimal case. ***: $p \le 0.001$, **: $p \le 0.01$, *: $p \le 0.05$, +: $p \le 0.1$, n.s. : $p > 0.1$

Figure 3A shows the median C-indices obtained for different thresholds with the elastic net penalty for BRCA. This methodology and representation allows optimal supervised and unsupervised thresholds with regard to the median C-index to be chosen for the pre-screening step (Fig. 3A, highlighted by a blue box). Figure 3B shows the number of genes screened by the IQR only, by the *p*-value of the single variable Cox model only, and by both.

Optimal thresholds vary according to the cancers. For example, for the elastic net penalty and C-index as a measure of prediction quality, the optimal thresholds for KIRP are 0.01 for the *p*-value of the single variable Cox model and 0 for the IQR (i.e. no pre-screening with the IQR), while they are 1 (i.e. no pre-screening with the *p*-value) and 2.5 for PAAD, respectively. We observe this diversity for the other penalties as well. A combination of supervised and unsupervised pre-screening is chosen in the optimal case with the C-index (resp. the IBS) for 7 (resp. 11) cancers for the ridge, 9 (resp. 13) cancers for the lasso, 6 (resp. 9) cancers for the elastic net, and 11 (resp. 13) cancers for the adaptive elastic net. Thus a combination of both supervised and unsupervised pre-screening techniques is valuable to remove irrelevant genes.

### The bi-dimensional pre-screening procedure increases prediction performance significantly for some cancers

Figure 3C shows improved prediction performance when the bi-dimensional pre-screening procedure is applied for BRCA. Without pre-screening, with ridge penalty, the median C-index for BRCA is $C = 0.651$ ($95\%$ confidence interval [0.629 ; 0.665]), improved to $C = 0.674$ ([0.657 ; 0.685]) with the optimal pre-screening. To note, for ridge and AEN, the optimal threshold for *p*-value is 1, meaning that all the genes are retained with the supervised procedure. Overall, the bi-dimensional pre-screening is at least neutral and at best improves the C-index, while removing irrelevant genes for prediction. The C-index is significantly increased after pre-screening for 3 cancers for the ridge, 8 cancers for the lasso, 7 cancers for the elastic net, and 5 cancers for the adaptive elastic net (Supplementary Fig. S8 and S9). The most important increase of C-index is observed for LAML and elastic net (0.026), but the typical improvement on C-index remains modest, around 0.015.

Similarly, the IBS is significantly decreased for 9 cancers for the ridge, 3 cancers for the lasso, 8 cancers for elastic net, and 11 cancers for the adaptive elastic net (Supplementary Fig. S10 and S11). The most important decrease on IBS is also observed for LAML, but with the adaptive elastic net (0.044).

Overall, the bi-dimensional pre-screening step leads to improved predictions, regardless of the penalty method, for LGG, KIRC, BLCA, LAML and BRCA with the C-index as the performance metric, and for UCEC, BLCA, LAML, BRCA, and PAAD with the IBS. For the other cancers, the improvement is method-dependent (e.g. for LIHC and the C-index, the median increase is 0.02 for the elastic net, but 0 for the ridge).

### Pre-screening prevents selection of irrelevant genes

For BRCA, 27 out of the 45 genes selected by elastic net (originally near 20,000 features) shows low variability among patients (i.e. $IQR < 1$, lower than the optimal threshold, Supplementary Fig. S12). Pre-screening on gene variability avoids the selection of these genes, for which the difference among patients are difficult to measure in practice.

However, all of these selected 45 genes have a corrected *p*-value below the optimal threshold (0.5). The pre-screening on corrected *p*-values allows to reduce the dimension (i.e. genes with corrected *p*-values greater than 0.5 are eliminated by screening), thus accelerating the computations. The genes selected by the penalization methods already have low *p*-values from single variable Cox model.

We observed these two properties for all datasets (data not shown): genes selected by lasso likes penalization have all low *p*-values, but not all a high *IQR*.

### Penalization methods provide comparable prediction performances after pre-screening

**Fig. 4:**
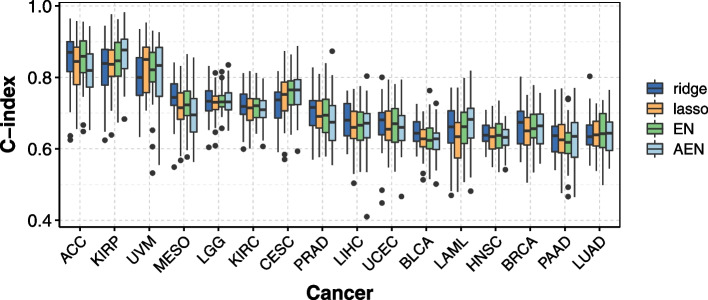
C-index obtained after pre-screening for the 16 cancers and the convex penalization methods studied (i.e. ridge, lasso, elastic net, adaptive elastic net). In each case, we computed the C-index by 10 repetitions of a nested 5-fold cross validation (section Suggested screening algorithm)

Figure 4 shows that overall, after the bi-dimensional pre-screening step, the performances of each penalization are similar, with small differences for a few cancer. We can in particular notice that in most cases EN does not provide better nor poorer performances than lasso. The prediction performance, however, strongly depends on cancer studied.

Among the noticeable differences, the ridge penalty reaches higher C-indices than the other penalizations for MESO and BLCA, but lower for CESC (Fig. 4). When focusing on the methods that allow the selection of a subset of genes (i.e. lasso, elastic net, adaptive elastic net), none outperforms the others. The lasso obtains poorer performances than the two others for LAML, and the elastic net dominates the adaptive elastic net for ACC and MESO.

Similarly, by choosing IBS as a metric, the ridge penalty performs poorer than the other penalizations for CESC (Supplementary Fig. S12). Among the selection methods, none dominate the others, and they perform very similarly. We can notice that the adaptive elastic net (resp. the lasso) performs slightly worse than the lasso and the elastic net (resp. the elastic net and the adaptive elastic net) for PRAD (resp. for LIHC). After the bi-dimensional pre-screening, the number of genes selected by the elastic net and the adaptive elastic net are comparable, and is twice as large as the one obtained with the lasso.

The two non-convex penalization methods lead to different performance: while SCAD performs similarly as the lasso-like methods, MCP clearly shows degraded performance with C-index, and to a less extent with IBS (Supplementary Fig. S14 and S15). More precisely, calculating the median C-index among the 50 C-indices for each penalization method, MCP obtains the worst performance for 12 out of the 16 cancers studied, while ridge reaches the best performance for 9 out of 16. For the IBS, AEN reaches the worst performance for 11 cancers, followed by MCP for 4 cancers. The best performances were again obtained by ridge (8 cancers) followed by lasso (5 cancers).

### Optimal bi-dimensional pre-screening shows improved performance on an independent dataset

**Fig. 5:**
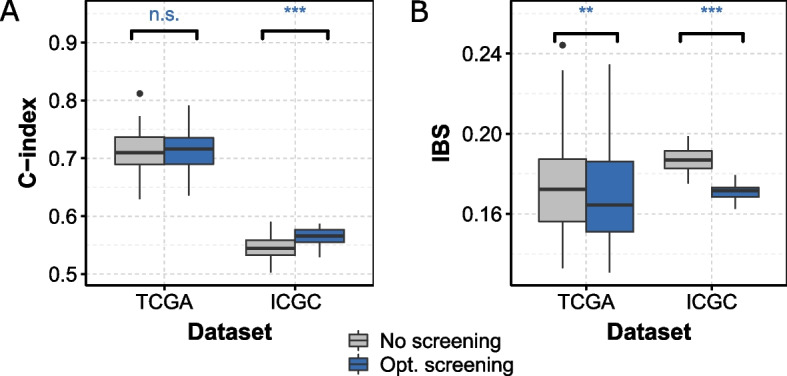
Performance of the predictions for ccRCC patients, test on two different datasets. In gray without pre-screening, in blue after optimal bi-dimensional pre-screening performed on the TCGA dataset. In both case, the model is tested on an independent set not used for learning, either TCGA or ICGC. (A) C-index metric, higher is better. (B) IBS metric, lower is better. In each case, we computed the C-index by 10 repetitions of a nested 5-fold cross validation (section Suggested screening algorithm). Paired Wilcoxon tests. ***: $p \le 0.001$, **: $p \le 0.01$, *: $p \le 0.05$, +: $p \le 0.1$, n.s. : $p > 0.1$

Figure 5 shows the performance of the model on the same TCGA dataset through cross-validation, and on an independent ICGC:RECA dataset. First, we observe on the independent dataset a strong reduction in performance on the C-index, which we interpret by the small proportion of patients with aggressive cancer in ICGC:RECA (14% of clinical stage III and IV patients in ICGC, compared to 39% in TCGA). Interestingly, even though there is almost no improvement in the C-index after pre-screening procedure is applied on TCGA (from $C = 0.71$ [0.702 ; 0.72] to $C = 0.716$ [0.698 ; 0.730]), the improvement is clear when the model learned on TCGA datase,t after the pre-screening procedure, is applied on the ICGC dataset ( (from $C = 0.545$ [0.535 ; 0.553] to $C = 0.566$ [0.560 ; 0.573]), Fig. 5A). Besides, the improvement is clear and strong when measuring the performance with the IBS, both on TCGA as a test set, and on ICGC (Fig. 5B): for TCGA:KIRC from $IBS = 0.172$ [0.165 ; 0.18] to $IBS = 0.164$ [0.159 ; 0.172], and for ICGC:RECA from $IBS = 0.187$ [0.185 ; 0.19] to $IBS = 0.172$ [0.170 ; 0.172].

### Simulations show improved IBS performance after pre-screening

Supplementary Fig. 16 shows the C-index obtained for the simulated models, before pre-screening and with optimal pre-screening. The C-index performance strongly depends on the simulation runs (with a median C-index being 0.8, 0.73 or 0.65 for simulations 1 to 3). It suggests that the vector of $\beta$ values strongly impacts the predictability of a given dataset. Similarly to the real TCGA dataset (Fig. 5), the pre-screening does not improve nor degrade the C-index of the model learnt.

Supplementary Fig. 17 shows the IBS obtained for the simulated models, before pre-screening and with optimal pre-screening. Also, the IBS performance strongly depends on the simulation runs (with a median IBS being 0.21, 0.11 or 0.12). Unlike the C-index, IBS is improved by the pre-screening procedure, also in line with what we observe for the real TCGA dataset (Fig. 5).

On each of the 3 simulated datasets, we also pre-filtered correlated genes, to keep only one gene in a group of correlated ones (with a correlation coefficient above 0.5 in absolute value, we kept the most correlated to survival in a single variable Cox model). This led to a reduction of the number of genes, from 17,249 to 4,890. Some of the suppressed genes were in the ground truth set, used to simulate the data. However, the performance of the models were equivalent with both sets of genes, with and without pre-screening, in the two metrics investigated (C-index and IBS, not shown). We hypothesize that the explanation lies in the fact that the lasso-like penalizations also select only one variables among correlated ones [20].

### Bi-dimensional pre-screening outperforms ISIS for prediction

First, it is worth noting that screening genes with individual correlation with survival using $|\beta |$ coefficients or *p*-values of a single variable Cox model are almost equivalent procedures. The correlation between the former and the latter are above 0.99 for all cancers (Supplementary Fig. S18). In that sense, pre-screening on *p*-values only is very close to pre-screening on $|\beta |$ coefficients. The *p*-values have the advantage of taking into account the uncertainty associated with the estimation of the coefficients and facilitate the choice of the thresholds in comparison with the beta coefficients. Second, for most cancers, pre-screening on gene variability among patients by setting a threshold on IQR leads to higher prediction performances. However, SIS (without iterations) and ISIS (with iterations) algorithms focuses only on individual correlation with survival.

**Fig. 6:**
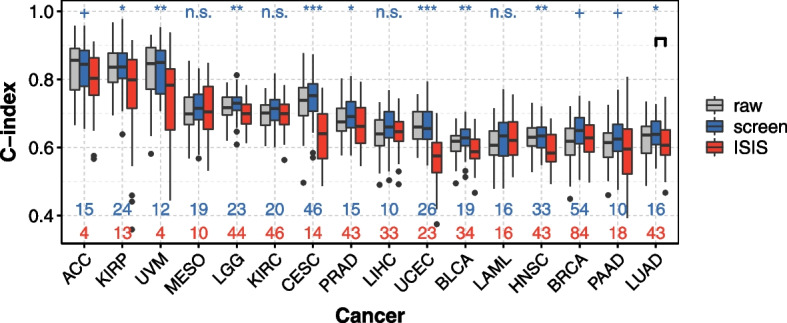
C-index obtained with the lasso without pre-screening (gray), with the lasso after the bi-dimensional pre-screening (blue), and with independent sure independence screening (ISIS, red). We computed the C-indices by 10 repetitions of a K-fold-validation (K=5). As an indication, the *p*-values obtained after a Wilcoxon test between ISIS and the bi-dimensional pre-screening (stars above the graphics) are provided. The 16 *p*-values are corrected with Benjamini-Hochberg method. Blue stars: the median C-index is lower for the bi-dimensional pre-screening. Blue numbers: number of genes selected by the lasso after bi-dimensional pre-screening. Red numbers: number of genes retained by ISIS. ***: $p \le 0.001$, **: $p \le 0.01$, *: $p \le 0.05$, +: $p \le 0.1$, n.s. : $p > 0.1$

Second, we observe better prediction performances with our bi-dimensional pre-screening algorithm compared with ISIS procedure. The C-indices are significantly higher for 9 of the 16 cancers studied (Fig. 6). For IBS, the results are equivalent for all cancers, with the exception of CESC for which the IBS is significantly smaller with bi-dimensional pre-screening (Supplementary Fig. S19).

Finally, the number of genes removed by the ISIS procedure is much greater than by our bi-dimensional pre-screening algorithm. For example, the median number of genes retained by the bi-dimensional pre-screening for BRCA with regard to the C-index is 1039 for the elastic net (Fig. 3B), and 84 for the ISIS (Fig. 6). However, the penalization methods (i.e. lasso, elastic net, adaptive elastic net) make it possible to further reduce the number of genes after the pre-screening step, down to 54 for BRCA and lasso (Fig. 6).

### Pre-screening without any cross-validation procedure

Previous sections show the interest of the cross-validation pre-screening procedure to decrease in a controlled manner the number of variables before further selection with lasso like procedures. If the number of events is too small to perform a cross-validation, and/or the required CPU is too high for the number of variables used to learn the model, we herein investigate whether general rules can be used for all cancers. We noticed in a previous section that the variables selected using a lasso like penalized Cox model are also individually correlated with patient survival, meaning that they all have a small *p*-value in a single variable Cox model. This property is not true for *IQR*. Besides, higher is the number of patients in a cohort, lower will be the *p*-value for a given variable: so, we will not use a threshold on *p*-values to provide a general advice. We thus decided to investigate to which extent pre-selecting a given number of genes with the lowest *p*-values would degrade the performance of the model for all cancers. Again in this section, we use the cross validation framework described Fig. 1 to avoid using data twice for learning and evaluation. Supplementary Figs. S20-23 show that using the top 50 genes in terms of *p*-values lead to reduced performances for CESC, MESO and KIRP cancers, which is especially true for C-index. However, when pre-selecting on the top 200, 500 or 2,000 genes, the performance are similar, while reducing drastically the number *p* of variables.

## Discussion

Like many others but in a complementary way [6, 7, 66], our work shows the benefit of using mRNA-seq data of the tumor together with clinical variables for typically half of the cancer subtypes for which mRNA profiles carry prognostic power. However, we restricted the clinical variables to common ones, shared for most cancer subtypes. Cancer-specific clinical variables exist (e.g. estrogen receptor status for breast cancer) and can further improve prediction obtained with clinical variables. Thus, the general conclusions we reached could be balanced for given cancer subtypes with the use of selected cancer-specific variables. It nevertheless confirms the interest of tumor profiling for prediction. Besides, we have not modified the prognostic index to account for clinical variables. Thus, there remains a potential of improvement in integrating clinical variables and mRNA data which is beyond the scope of the present work.

Then, as others before [7, 67], we confirm here that the overall survival prediction quality is cancer dependent, and it can be as low as a pure random prediction, with a median concordance of 0.51 for ESCA (Supplementary Fig. S2). Different hypotheses can be drawn up to explain these differences across cancers. First, many studies show intra-tumoral heterogeneity (e.g., genetic and phenotypic variations across different geographic regions for one tumor subtype) [68]. The expression levels of the genes correlated with survival (the input of the Cox model) might thus vary across spatial area of the tumor, although the survival time of the patients is a global outcome. Secondly, other explanatory variables might better predict overall survival for some cancers. For example, [69] recently showed that tumor microbiome diversity influences patient outcome for pancreatic cancer.

The existence of subtypes of cancers with different molecular characteristics [70] would lead to poor regression performance and prediction, as here all of the patient data were processed with the same model. Thus the integration of new biological data, and the consideration of hidden variables (e.g. tumor purity, [63]) could improve the performance of the models.

Then, the unsupervised pre-screening step allows genes that show the highest expression variability among patients to be retained. However, the main issue of pre-screening with IQR (i.e., the difference between the 75th and 25th percentiles) does show up when fewer than 25% of the patients express a gene leading to poor prognosis. In this case, the gene would not be kept by the pre-screening step, while it could be important for prediction. Other metrics can be used to overcome this issue; e.g., the IQR can be easily replaced by the difference between the 90th and 10th percentiles using the same methodology.

We applied the pre-screening procedure only on transcriptomic data. When performing a supervised pre-screening on combined clinical and transcriptomic data, it could be interesting to test on both individual genes and clinical data, in order to pre-select only genes that have additional prognostic values than clinical data.

In this study, we have not investigated how to group genes following current knowledge, for example clinically relevant genes. Also, other penalizations could be used to account for pathway information [71]. In three simulated datasets, we have pre-filtered the genes, to keep only one in a group of correlated genes, choosing an arbitrary threshold of 0.5. This procedure is interesting as it highly decreases the number of genes with low computation time, except if one performs a cross-validation procedure to fix the threshold.

In order to compare the prediction performances among different conditions, we performed a Wilcoxon test among the 50 metric values obtained after 10 repetitions of a K-fold (K=5) cross-validation. We have to warn that the *p*-value we provide is only indicative, as a real but small difference in two distributions can lead to arbitrarily small *p*-values when the number of repetitions tends to infinity. Besides, the different repetitions, using the same initial set of patients are not independent as samples are taken repeatedly from the same data set for several Monte-Carlo runs during the cross-validation procedure. For these reasons, we set a reasonable number of repetitions. However, the indicative *p*-value has the advantage to help the readability of the figures by focusing on most interesting box plots.

In order to apply predictions to patients in clinics, it is important to pursue research on how to normalize the data. While in the cross-validation procedure we provide a fair standardization, using the mean and standard-deviation of the expression of each gene in the learning set, this procedure did not work for the ccRCC independent dataset: the average and standard deviation were too different between the two experiments, probably due to the difference in protocols. Using the learning set parameters did not affect the C-index, but it clearly decreased IBS performance (not shown). To reach good performance, we had to use the mean and standard deviation of the test set. This is not practical in a clinical context. Thus, further improvements are required on the normalization and standardization of protocols, but this is beyond the scope of the present article. A hypothesis would be to use carefully selected normalizing genes, and to work on standardizing the sample preparation.

## Conclusions

To the best of our knowledge, this is the first study to gather 16 different cancer subtypes to evaluate various Lasso-like penalized Cox models together with 2 pre-screening procedures with 2 common metrics, namely C-index and IBS. In the context of ‘ultrahigh-dimensional data’ [14], we propose to pre-screen genes based on a robust estimation of their expression variability among patients and individual correlation with survival. This methodology and the proposed representation allow for: (i) the definition of the ‘optimal thresholds’ with rational justifications; (ii) the drastic reduction of the number p of features by removing irrelevant genes (i.e. not associated to survival or noisy) and mitigating the ‘curse of dimensionality’ in the multivariable Cox model; and (iii) optimization of the performance metrics of interest (i.e. C-index or IBS in this study). We also provide a benchmark of different penalization methods (i.e. ridge, lasso, elastic net, adaptive elastic net) after our bi-dimensional pre-screening procedure.

First, we showed that integration of mRNA-seq data with clinical data allows to improve predictions over clinical data alone. Second, we showed that our bi-dimensional pre-screening algorithm allows to improve predictions for some cancers regarding both the C-index (LGG, KIRC, BLCA, LAML and BRCA) and the IBS (UCEC, BLCA, LAML, BRCA, and PAAD), while removing irrelevant genes for prediction and for clinical and research applications. For the other cancers, the improvement depends on the chosen penalization, but can only be beneficial. Third, we demonstrated that the different penalization methods reached similar prediction performances, with slight differences among cancers. Fourth, our bi-dimensional pre-screening procedure allows to get higher C-indices than ISIS algorithm for 12 of the 16 cancers studied, and comparable for the 4 others. Finally, we have proved that the proposed methodology is valuable on an independent dataset. However, we have to acknowledge that the improvements remain modest. Other procedures than ISIS have been recently proposed, but are not yet implemented, nor the code provided with the original article [72, 73]. A benchmark among all these procedures would be an interesting perspective.

As practical conclusions, we first advise that the genes are pre-screened with both supervised and unsupervised approaches, with several (e.g., 10 in our study) repetitions of K-fold cross-validation (K=5) to calculate the optimal thresholds. If this procedure is not feasible, for example when integrating various omics data, selecting the top 200 to 2,000 mRNAs in terms of single variable Cox model *p*-value works well for all the datasets and methods we have investigated and allow to get effectively rid of irrelevant genes. Second, we advise equally the use of the ridge, the elastic net or the lasso penalization after the pre-screening step as they require lower computational time than the adaptive elastic net penalization. However, if a more parsimonious model is needed, we advise the use of lasso penalization, as it selects fewer genes to build the final risk score, without reducing much the prediction performance.

## Supplementary Information

**Additional file 1.** A document containing supplementary materials.

**Additional file 2.** A document containing supplementary Table 1 with TCGA cancer abbreviations.

**Additional file 3.** A document containing supplementary Figures 1-23 including the corresponding legends.

## Data Availability

The clinical and mRNA-seq TCGA datasets were obtained using the Broad GDAC FIREHOSE utility (https://gdac.broadinstitute.org). The code used to produce all the figures is shared at the following github repository https://github.com/remyJardillier/Survival_preScreening.
