# Supplementary material for: Prognosis of lasso-like penalized Cox models with tumor profiling improves prediction over clinical data alone and benefits from bi-dimensional pre-screening
Source: BMC Cancer. 2022 Oct 5;22:1045. doi: 10.1186/s12885-022-10117-1 (PMC9533541; doi:10.1186/s12885-022-10117-1)
Supplement: Supplementary file 1 — Additional file 1. A document containing supplementary materials. [file 12885_2022_10117_MOESM1_ESM.pdf]

Supplementary Materials

1 More details on the Cox model

The function L is called the 'pseudo-likelihood', because it is not a product of density functions, but a product of conditional probabilities. $\hat{\boldsymbol{\beta}}$ is computed by maximizing this pseudo-likelihood function: $\hat{\boldsymbol{\beta}} = \arg\max_{\boldsymbol{\beta}}(l(\boldsymbol{\beta}))$, with $l(\boldsymbol{\beta}) = \log(L(\boldsymbol{\beta}))$, the log-pseudo-likelihood.

Note that the Cox model is not intuitive, in the sense that it links genetic data to patient survival in an indirect way, through the hazard function. However, Cox pseudo-likelihood allows censored data to be efficiently dealt with. Moreover, this yields a robust inference procedure where the baseline function $h_0(t)$ does not need to be modeled or estimated in a parametric way. Finally, the estimation procedure leads to a convex optimization problem, for which efficient procedures and packages exist for computing $\hat{\boldsymbol{\beta}}$ [Friedman et al., 2010].

2 More details on the penalization methods

The ℓ_1 norm forces some coefficient estimates $\hat{\beta}_j, j = 1, ..., p$ to be zero, and allows the selection to be made. For multivariate Cox selection models, the genes selected are defined as the genes with nonzero $\hat{\beta}_j$ coefficients. It has been empirically observed that if there are high correlations between predictors, the ridge penalty provides better prediction performance than the lasso [Tibshirani, 1997], and that the shrinkage effect of the lasso is too strong for large effects [Zou, 2006]. The elastic net and the adaptive elastic net penalties have been developed to tackle these two issues, respectively.

We computed the weight of the penalty, λ, by K-fold cross-validation (K = 5) using the package *glmnet* [Friedman et al., 2010] in R version 3.6.0 [R Core Team, 2019]. The weight λ that minimizes deviation in the cross-validation is given by λ_{min}. We chose $\alpha = 0.3$ in the elastic net, as the deviance remains stable for different values of α and the number of genes selected starts to stabilize from this value (Supplementary Fig. S1). This choice makes it possible to maximize the effects of the ridge penalty while selecting a subset of genes.

For more details of the mathematical concepts used in this article, we refer the reader to the book 'The Statistical Analysis of Failure Time Data' [Kalbfleisch and Prentice, 2011].

References

[Friedman et al., 2010] Friedman, J. et al. (2010). Regularization paths for generalized linear models via coordinate descent. *Journal of Statistical Software*, 33(1):1–22.

[Kalbfleisch and Prentice, 2011] Kalbfleisch, J. D. and Prentice, R. L. (2011). *The Statistical Analysis of Failure Time Data*. AMLBook.

[R Core Team, 2019] R Core Team (2019). *R: A Language and Environment for Statistical Computing*. R Foundation for Statistical Computing, Vienna, Austria.

[Tibshirani, 1997] Tibshirani, R. (1997). The lasso method for variable selection in the cox model. *Statistics in Medicine*, 16(4):385–395.

[Zou, 2006] Zou, H. (2006). The adaptive lasso and its oracle properties. *Journal of the American Statistical Association*, 101(476):1418–1429.
