# Supplementary material for: Prognosis of lasso-like penalized Cox models with tumor profiling improves prediction over clinical data alone and benefits from bi-dimensional pre-screening
Source: BMC Cancer. 2022 Oct 5;22:1045. doi: 10.1186/s12885-022-10117-1 (PMC9533541; doi:10.1186/s12885-022-10117-1)
Supplement: Supplementary file 3 — Additional file 3. A document containing supplementary Figures 1-23 including the corresponding legends. [file 12885_2022_10117_MOESM3_ESM.pdf]

Supplementary Figures

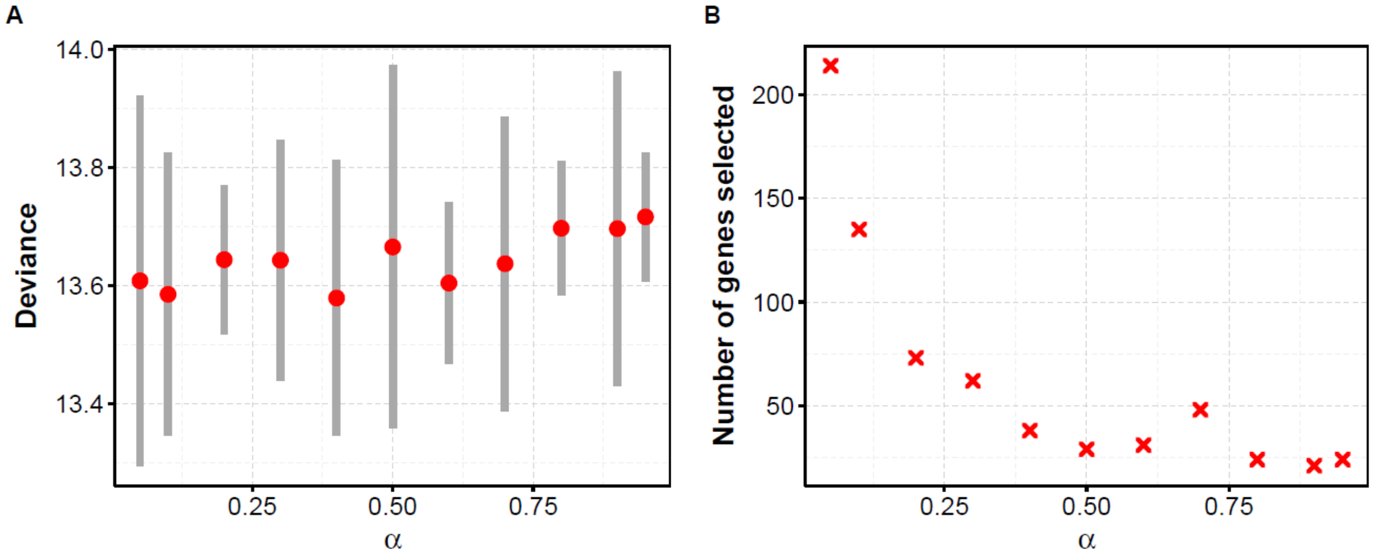

Supplementary Fig. S1. Deviance and number of genes selected for different values of α for BRCA.

We computed the deviance by K-fold cross validation (K=5) for each value of α.

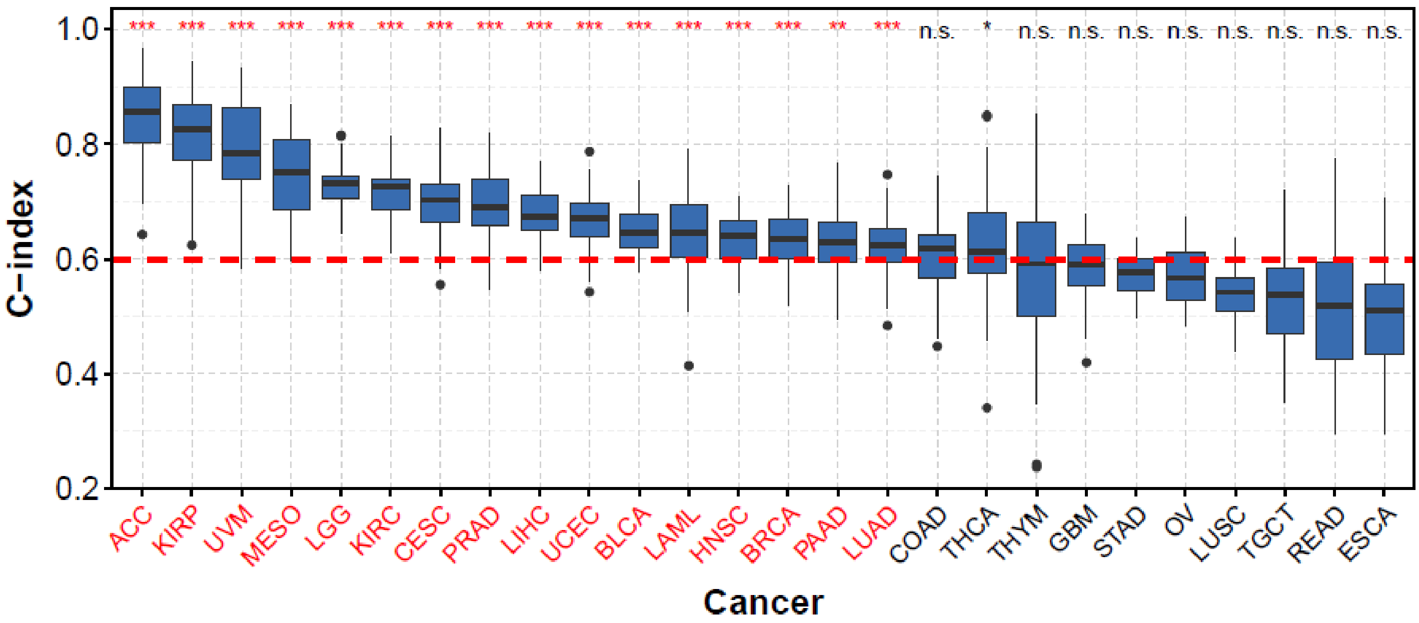

Supplementary Fig. S2. Boxplot of the C-indices computed by 10 repetitions of a K-fold cross validation (K=5) for all the 26 cancers.

We retained 16 cancers (red) that have a median C-index significantly above 0.6 according to a one-sided Wilcoxon test at level 0.01. We corrected the p-values with the Benjamini-Hochberg method (stars above the graphics).

Red dotted horizontal line : C-index of 0.6.

***: $p \leq 0.001$, **: $p \leq 0.01$, *: $p \leq 0.05$, +: $p \leq 0.1$, n.s. : $p > 0.1$

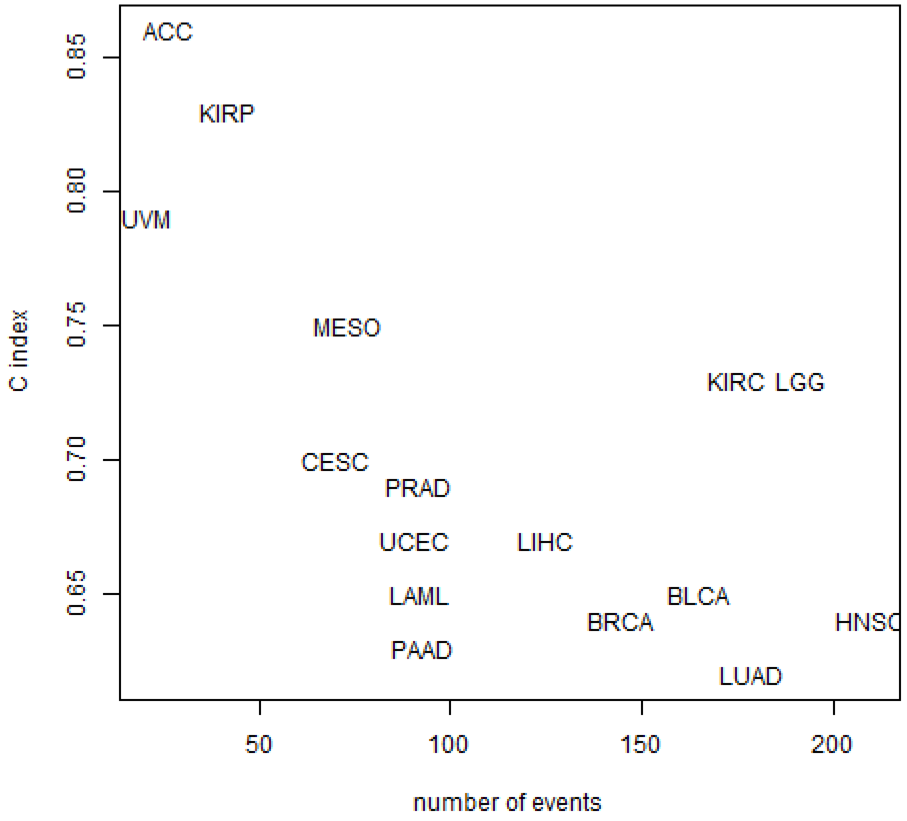

Supplementary Fig. S3. Median C-index (as indicated Table 1) as a function of the number of events per cancer subtype.

The codes for cancer subtypes are provided in Supplementary Tab. S1.

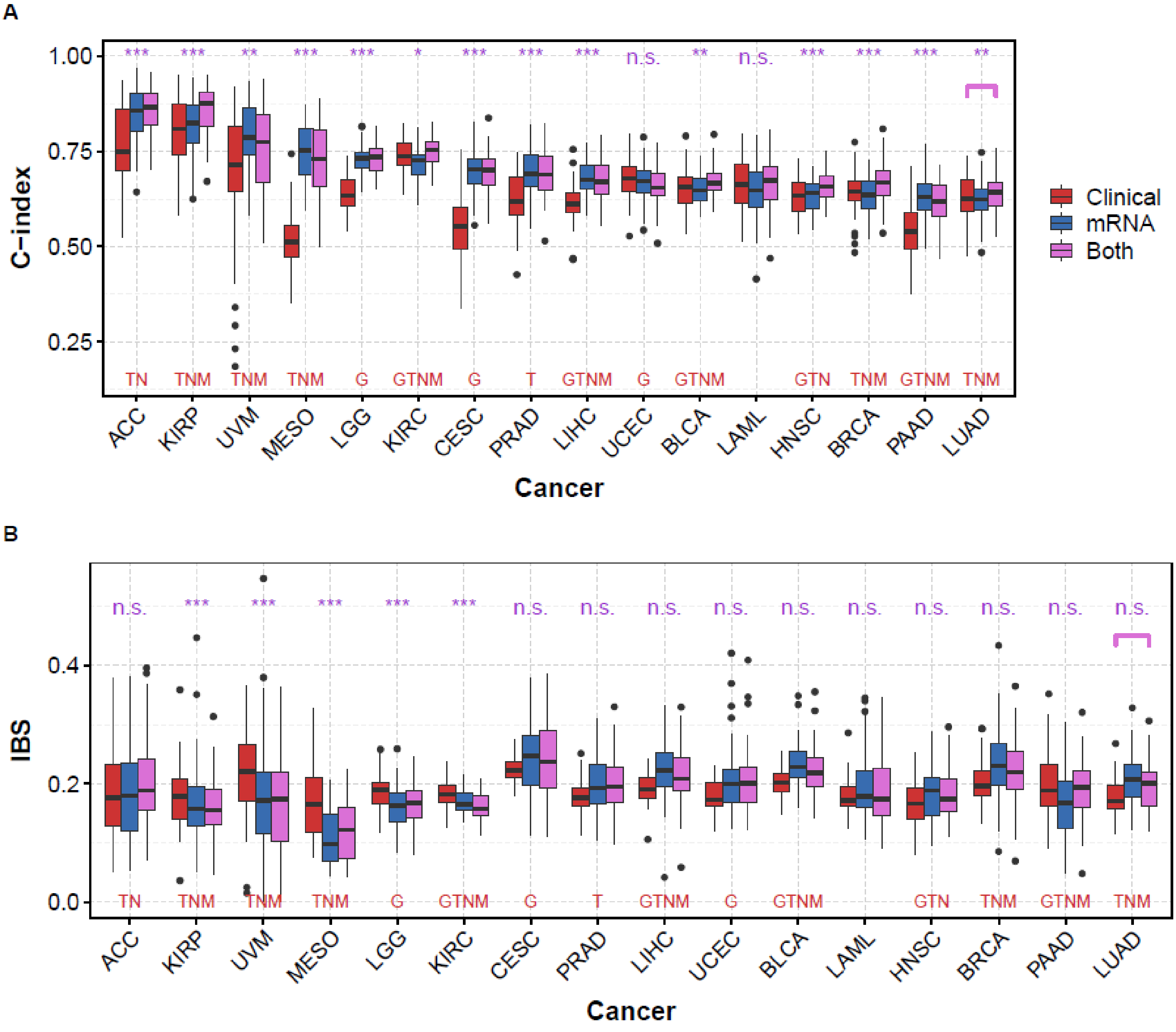

Supplementary Fig. S4. C-indices (A) and IBS (B) obtained with clinical data alone (red), mRNA-seq data alone (blue), and clinical and mRNA-seq data together (purple) for the 16 cancers studied in terms of pre-screening.

Procedure detailed in Figure 1. We computed the C-indices by 10 repetitions of a K-fold cross-validation (K=5). To evaluate whether the added value of mRNA-seq data for prediction is significant over clinical data alone, we computed the p-values of a one-sided Wilcoxon signed-rank test between clinical and both clinical+mRNA-seq (red versus pink boxplots, pink stars at the top of each graphic, Benjamini-Hochberg correction for the 26 p-values).

Red letters at the bottom of each graphics indicate the clinical data available (G: grade; T: tumor; N: node; M: metastasis). Age is available for all cancers, and gender only for non-unisexual cancers (CESC, PRAD, TGCT are sex-specific).

***: $p \leq 0.001$, **: $p \leq 0.01$, *: $p \leq 0.05$, +: $p \leq 0.1$, n.s. : $p > 0.1$

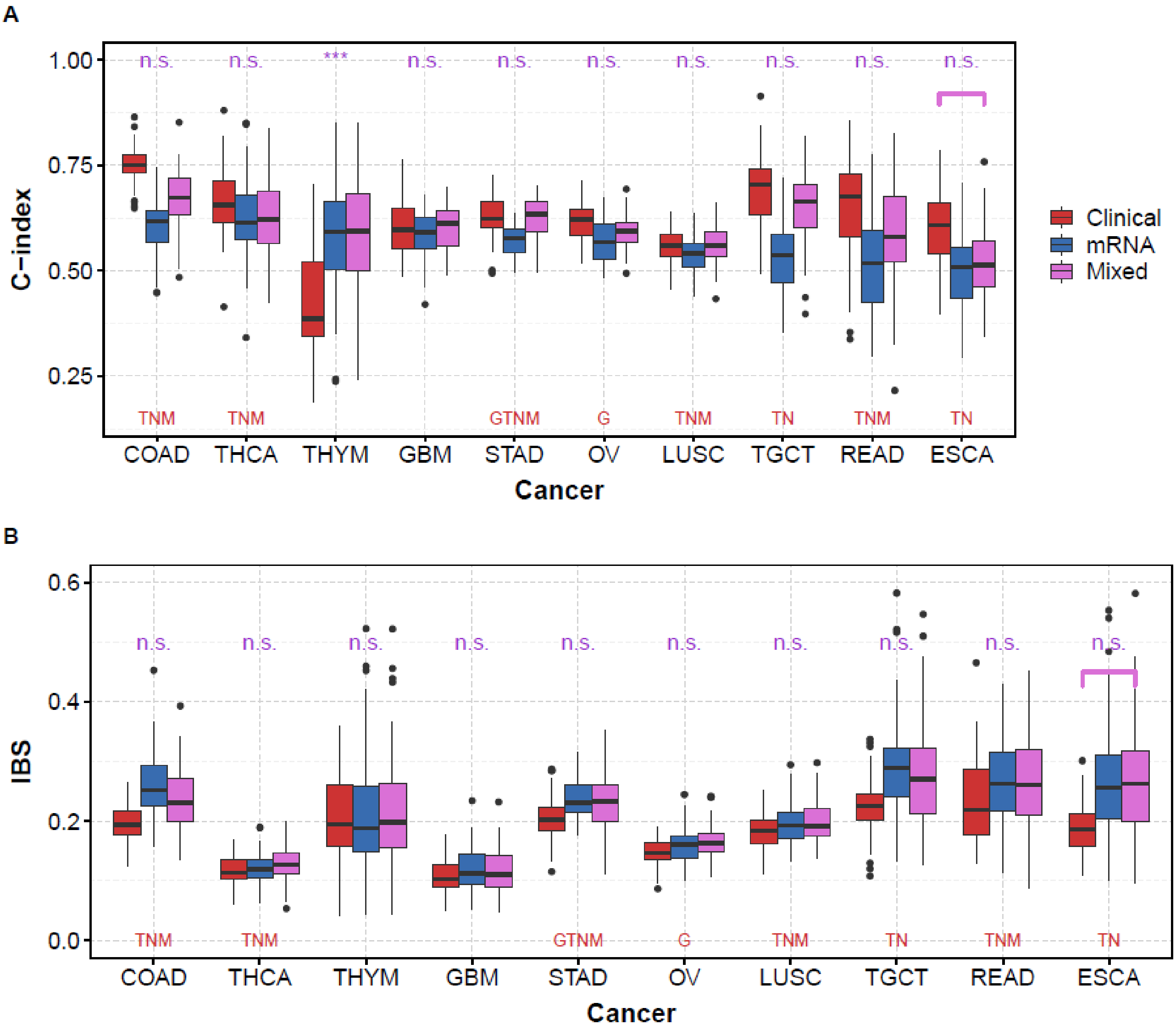

Supplementary Fig. S5. C-indices (A) and IBS (B) obtained with clinical data alone (red), mRNA-seq data alone (blue), and clinical and mRNA-seq data together (purple) for the 10 cancers not studied in terms of pre-screening.

Procedure detailed in Figure 1. We computed the metrics by 10 repetitions of a K-fold cross-validation (K=5). To evaluate whether the added value of mRNA-seq data for prediction is significant over clinical data alone, we computed the p-values of a one-sided Wilcoxon signed-rank test between clinical and both clinical+mRNA-seq (red versus pink boxplots, pink stars at the top of each graphic, Benjamini-Hochberg correction for the 26 p-values).

Red letters at the bottom of each graphics indicate the clinical data available (G: grade; T: tumor; N: node; M: metastasis). Age is available for all cancers, and gender only for non-unisexual cancers (CESC, PRAD, TGCT are sex-specific).

***: $p \leq 0.001$, **: $p \leq 0.01$, *: $p \leq 0.05$, +: $p \leq 0.1$, n.s. : $p > 0.1$

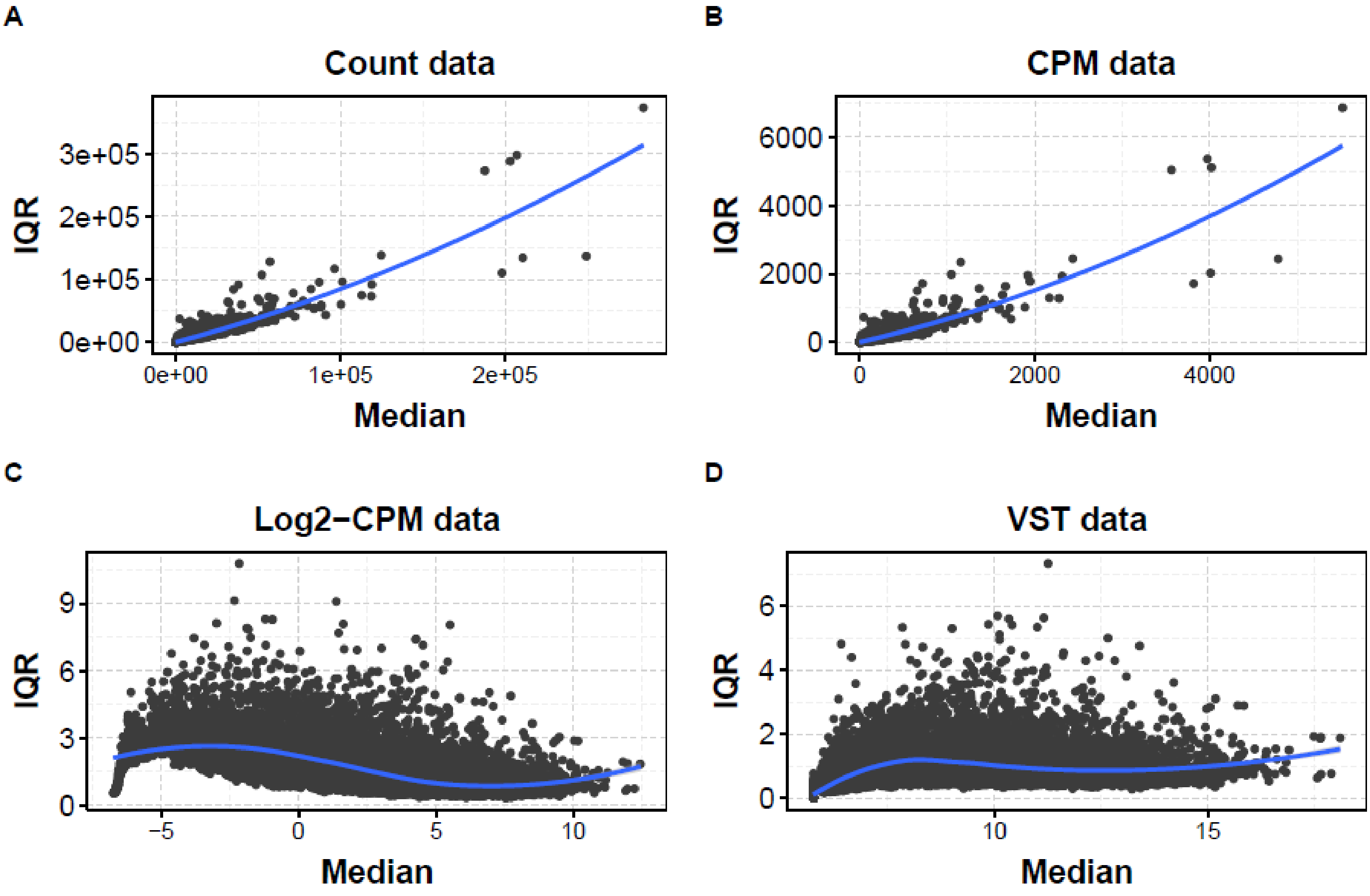

Supplementary Fig. S6. Median-IQR trend of gene expression for raw count data (A), CPM data (B), log2-CPM data (C), and VST data (D) for BRCA.

The blue curve is the loess estimate on each point (gene).

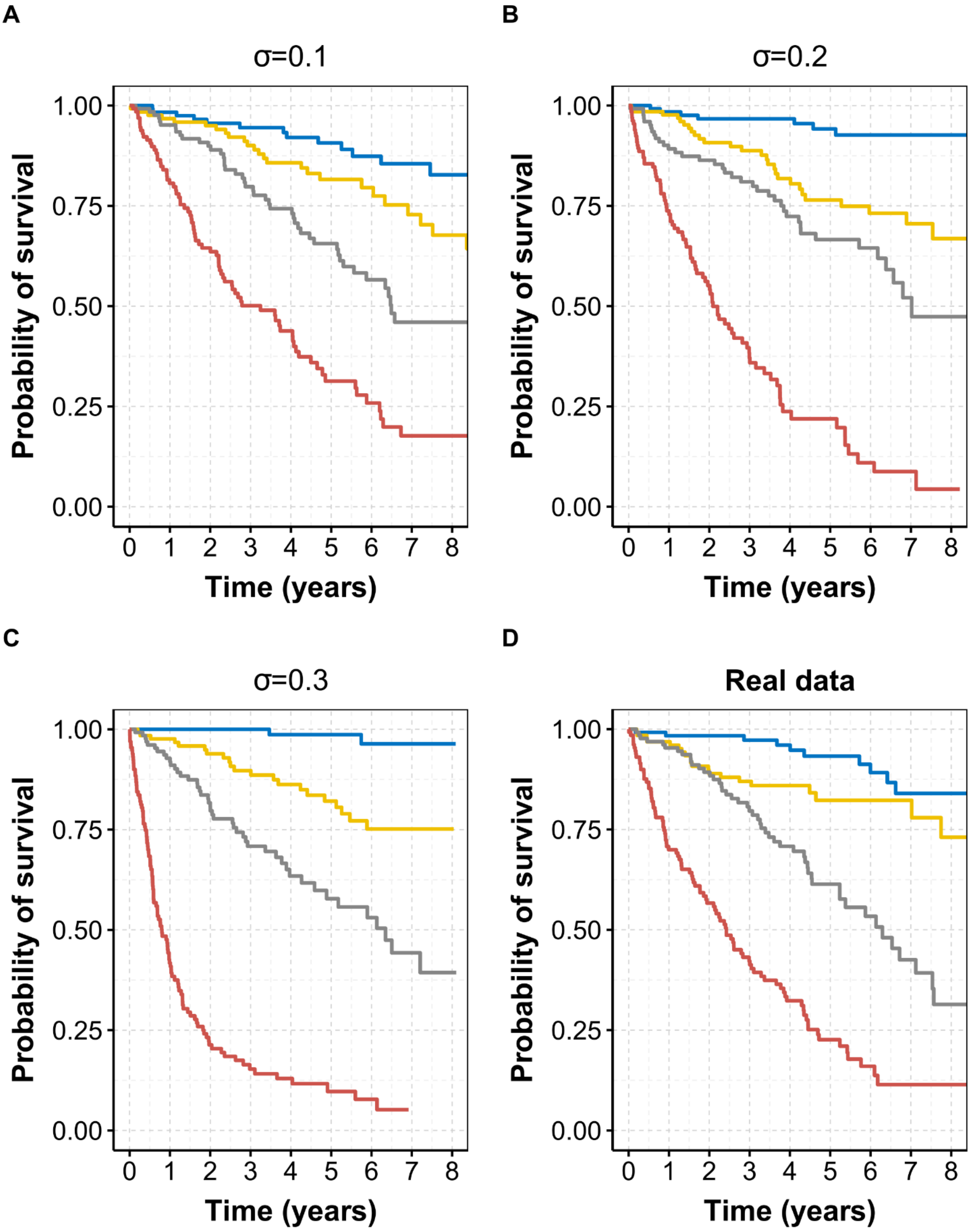

Supplementary Fig. S7. Kaplan-Meier curves of three simulations (A-C) with varying σ compared with the real TCGA dataset KIRC (D).

Simulated (A-C) or real (D) datasets are separated in 4 equal groups depending on prognostic indices (PI). σ corresponds to the standard used to simulate the β values in a centered normal law.

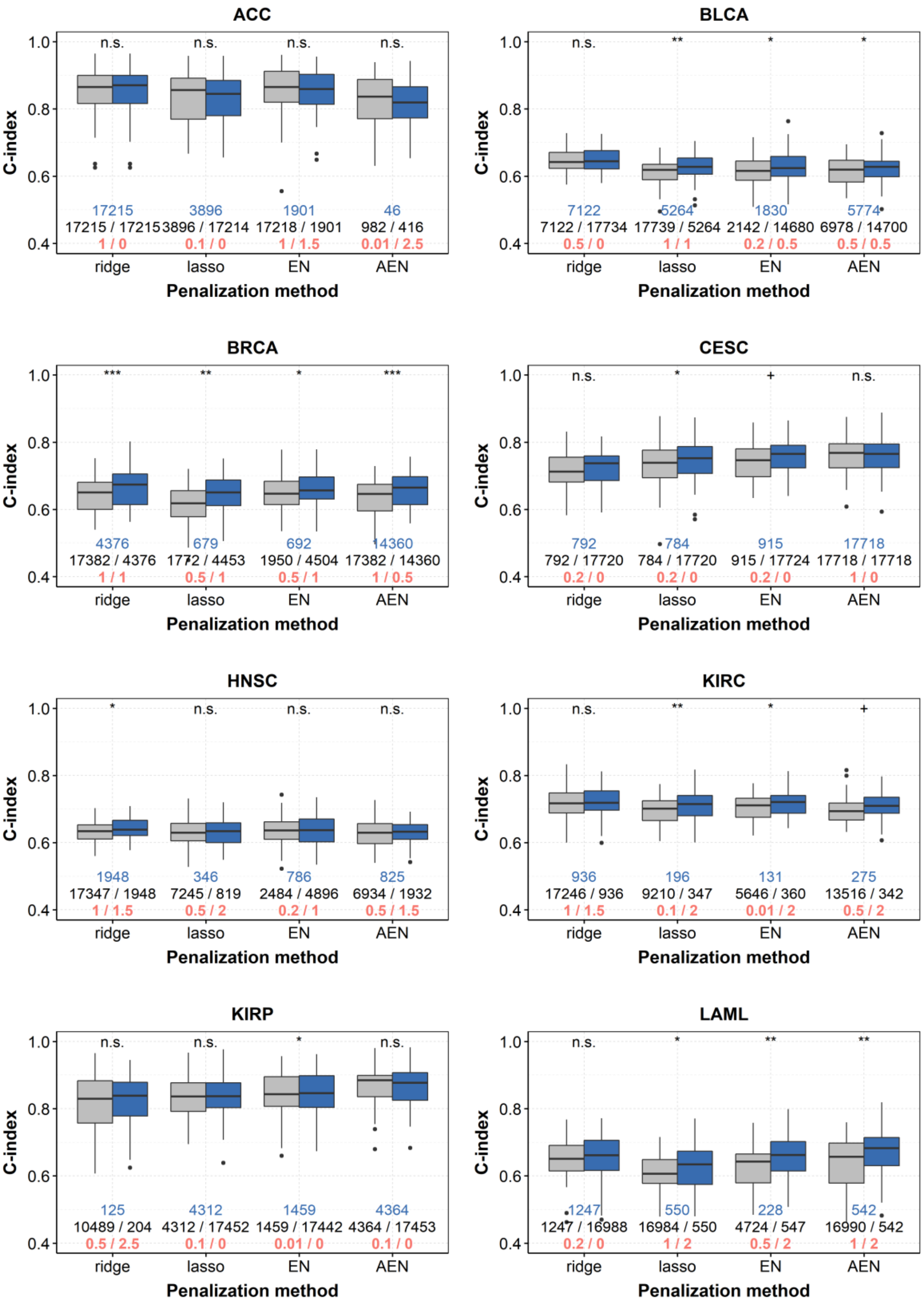

Supplementary Fig. S8. C-indices without pre-screening (gray boxplots) and with pre-screening in the optimal threshold (blue boxplots) for various Cox model penalizations applied to 8 cancers. Equivalent of Figure 2C.

Procedure detailed in Figure 1. Blue numbers are the number of genes retained after both thresholds of the prescreening step; black and red numbers are respectively the number of genes and the optimal thresholds retained by supervised (left) and unsupervised (right) pre-screening.

***: $p \leq 0.001$, **: $p \leq 0.01$, *: $p \leq 0.05$, +: $p \leq 0.1$, n.s. : $p > 0.1$

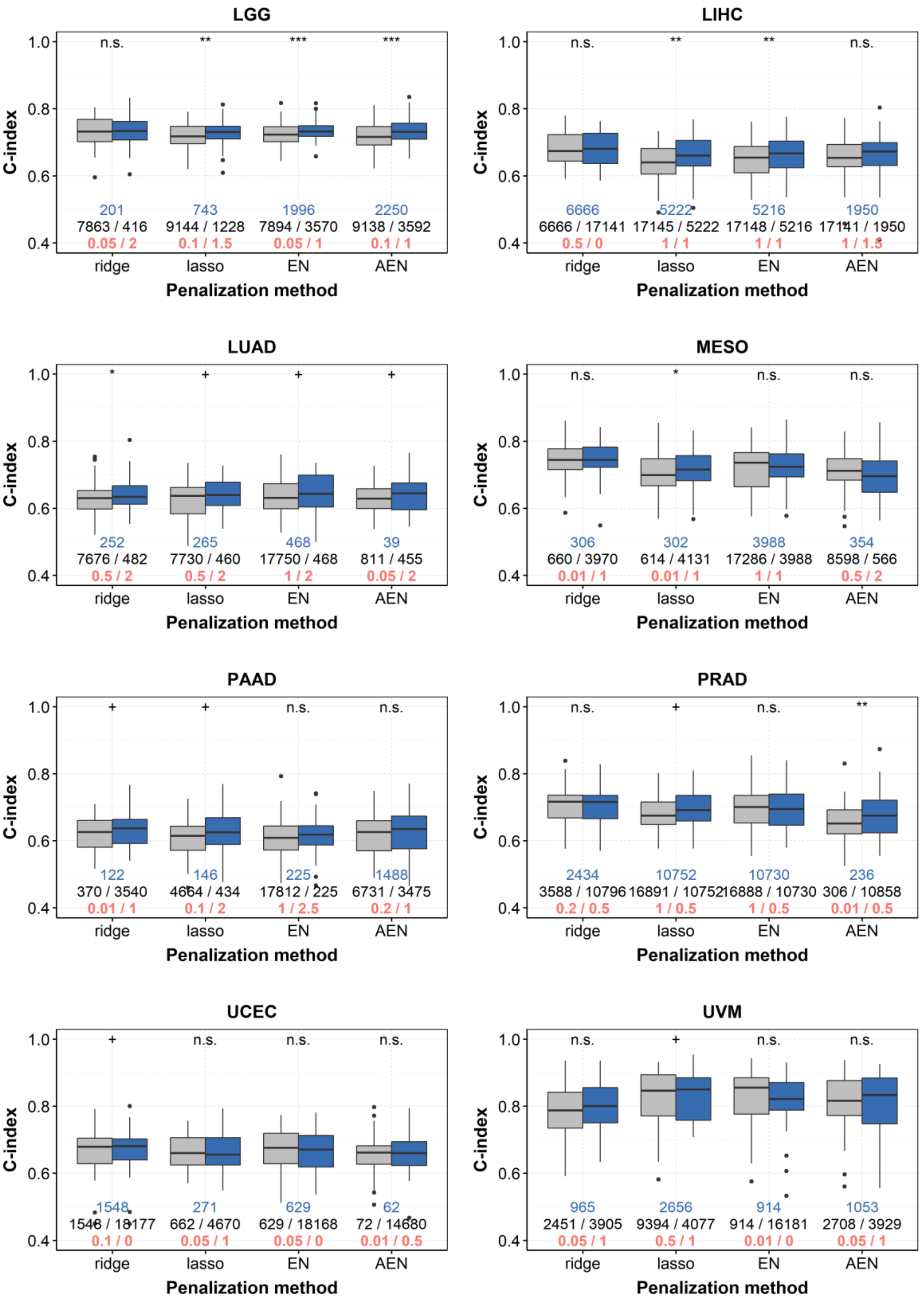

Supplementary Fig. S9. C-indices without pre-screening (gray boxplots) and with pre-screening in the optimal threshold (blue boxplots) for various Cox model penalizations applied to 8 other cancers. Equivalent of Figure 2C.

Procedure detailed in Figure 1. Blue numbers are the number of genes retained after both thresholds of the prescreening step; black and red numbers are respectively the number of genes and the optimal thresholds retained by supervised (left) and unsupervised (right) pre-screening.

***: $p \le 0.001$, **: $p \le 0.01$, *: $p \le 0.05$, +: $p \le 0.1$, n.s. : $p > 0.1$

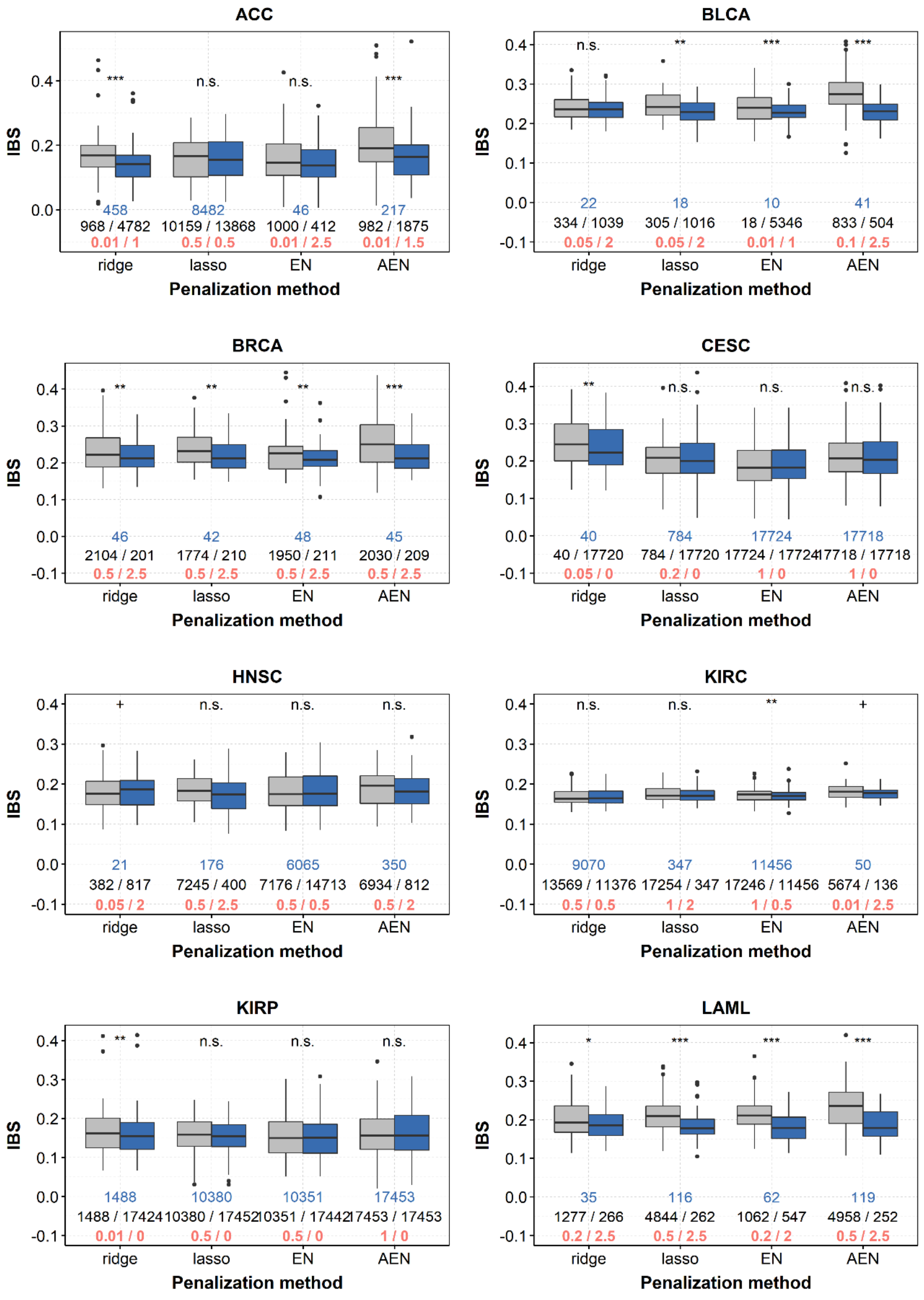

Supplementary Fig. S10. IBS without pre-screening (gray boxplots) and with pre-screening in the optimal threshold (blue boxplots) for various Cox model penalizations applied to 8 cancers. Equivalent of Figure 2C.

Procedure detailed in Figure 1. Blue numbers are the number of genes retained after both thresholds of the prescreening step; black and red numbers are respectively the number of genes and the optimal thresholds retained by supervised (left) and unsupervised (right) pre-screening.

***: $p \leq 0.001$, **: $p \leq 0.01$, *: $p \leq 0.05$, +: $p \leq 0.1$, n.s. : $p > 0.1$

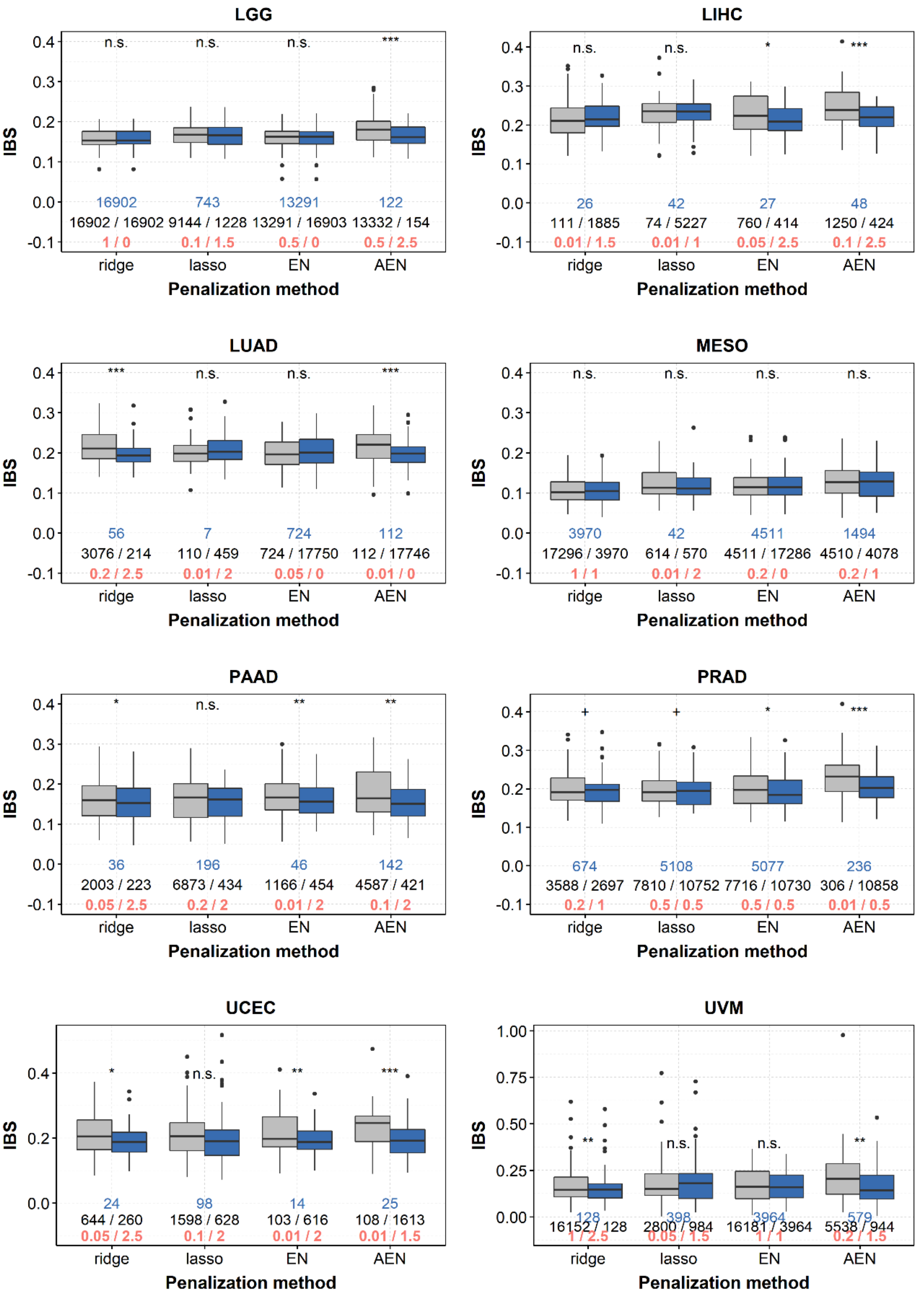

Supplementary Fig. S11. IBS without pre-screening (gray boxplots) and with pre-screening in the optimal threshold (blue boxplots) for various Cox model penalizations applied to 8 other cancers. Equivalent of Figure 2C.

Procedure detailed in Figure 1. Blue numbers are the number of genes retained after both thresholds of the prescreening step; black and red numbers are respectively the number of genes and the optimal thresholds retained by supervised (left) and unsupervised (right) pre-screening.

***: $p \leq 0.001$, **: $p \leq 0.01$, *: $p \leq 0.05$, +: $p \leq 0.1$, n.s. : $p > 0.1$

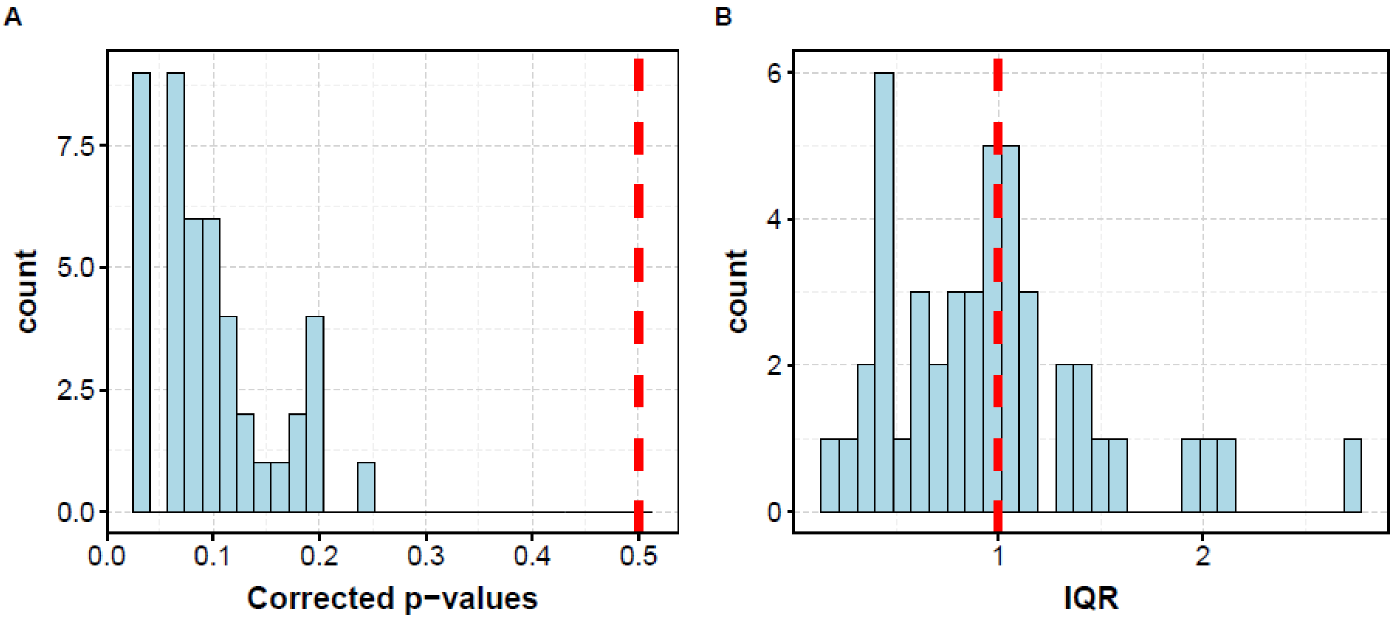

Supplementary Fig. S12. P-values of univariate Cox models corrected with the Benjamini-Hochberg procedure (A) and IQR of the VST data (B) of the genes selected by elastic net for BRCA (without pre-screening).

The red dashed vertical lines are the optimal thresholds for each case.

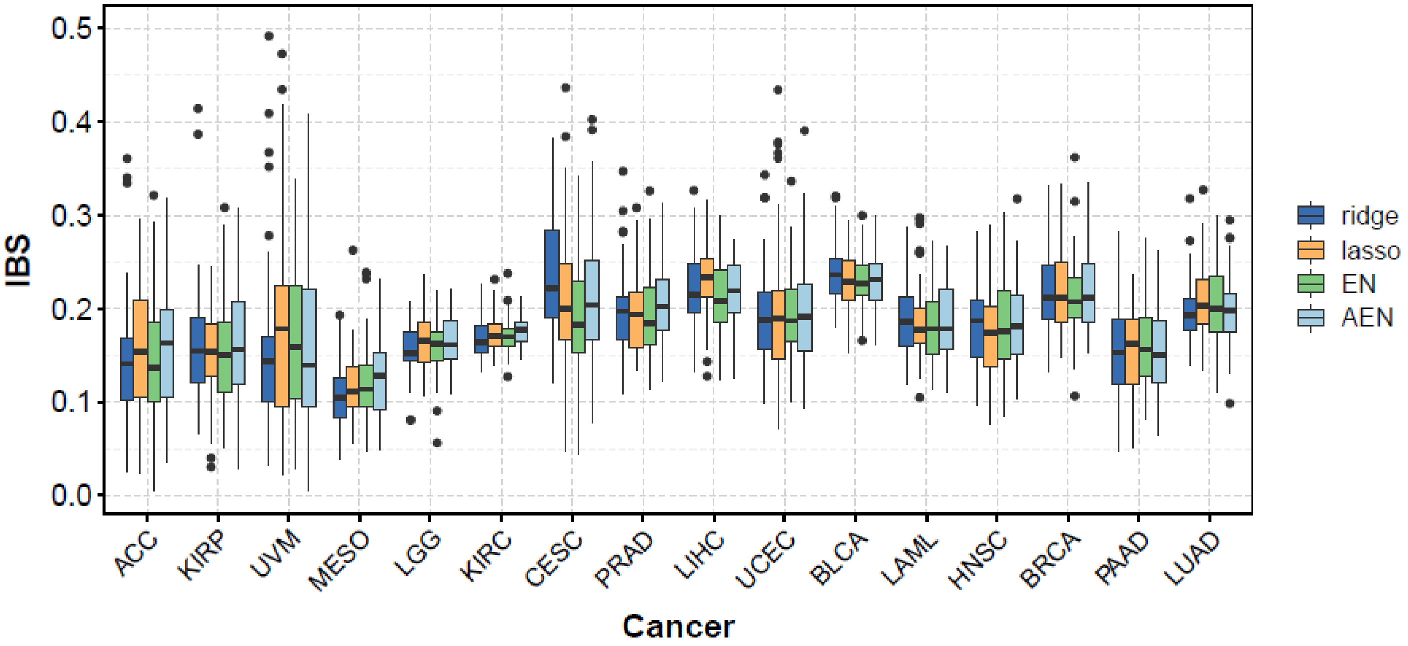

Supplementary Fig. S13. IBS obtained after pre-screening for the 16 cancers and the penalization methods studied (i.e. ridge, lasso, elastic net, adaptive elastic net).

Procedure detailed in Figure 1, that is in each case, we computed the IBS by 10 repetitions of a nested 5-fold cross validation.

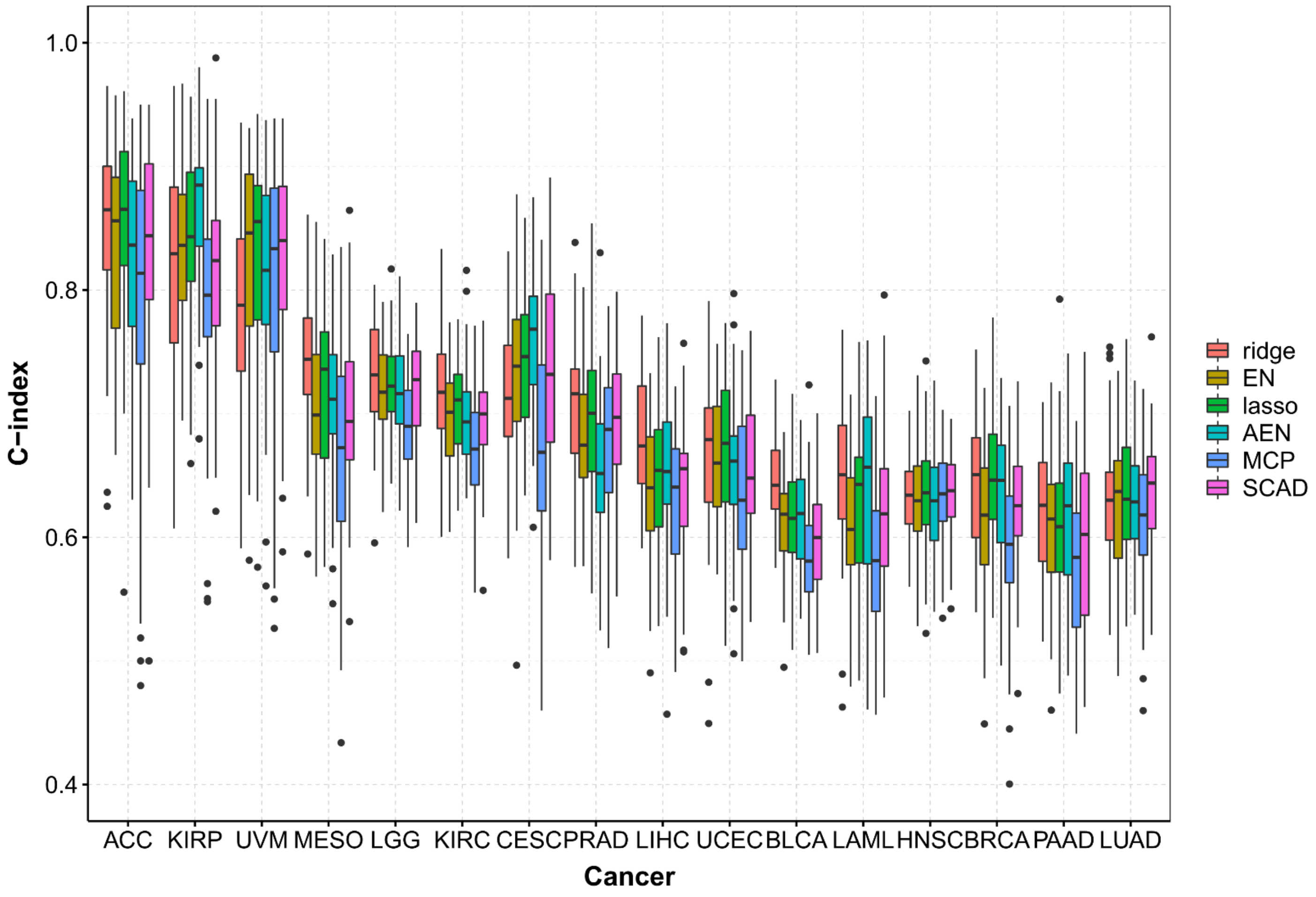

Supplementary Fig. S14. C-index obtained after pre-screening for the 16 cancers and the penalization methods studied (i.e. ridge, lasso, elastic net, adaptive elastic net) together with SCAD and MCP.

Procedure detailed in Figure 1, that is in each case, we computed the C-index by 10 repetitions of a nested 5-fold cross validation.

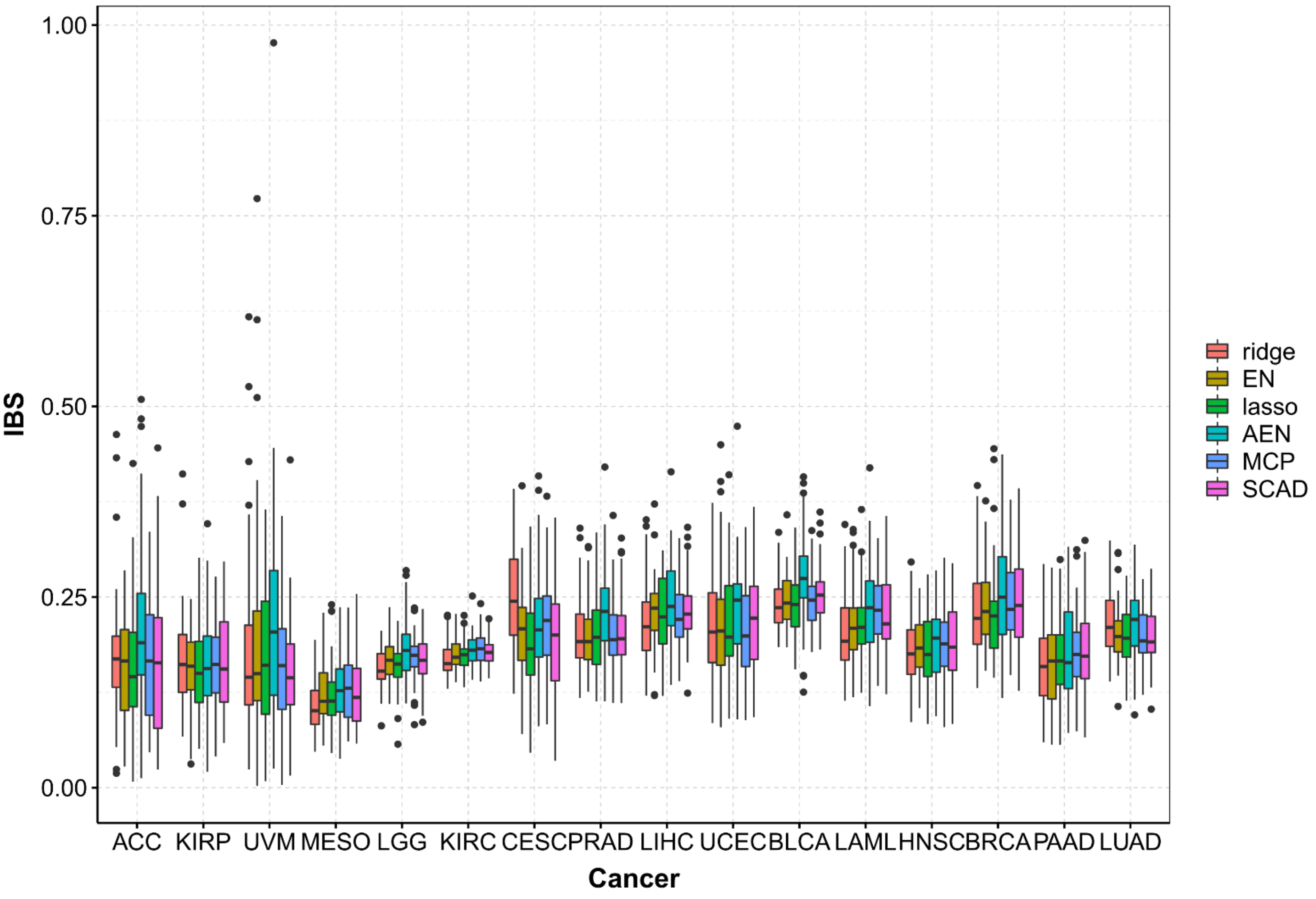

Supplementary Fig. S15. IBS obtained after pre-screening for the 16 cancers and the penalization methods studied (i.e. ridge, lasso, elastic net, adaptive elastic net) together with SCAD and MCP.

Procedure detailed in Figure 1, that is in each case, we computed the IBS by 10 repetitions of a nested 5-fold cross validation.

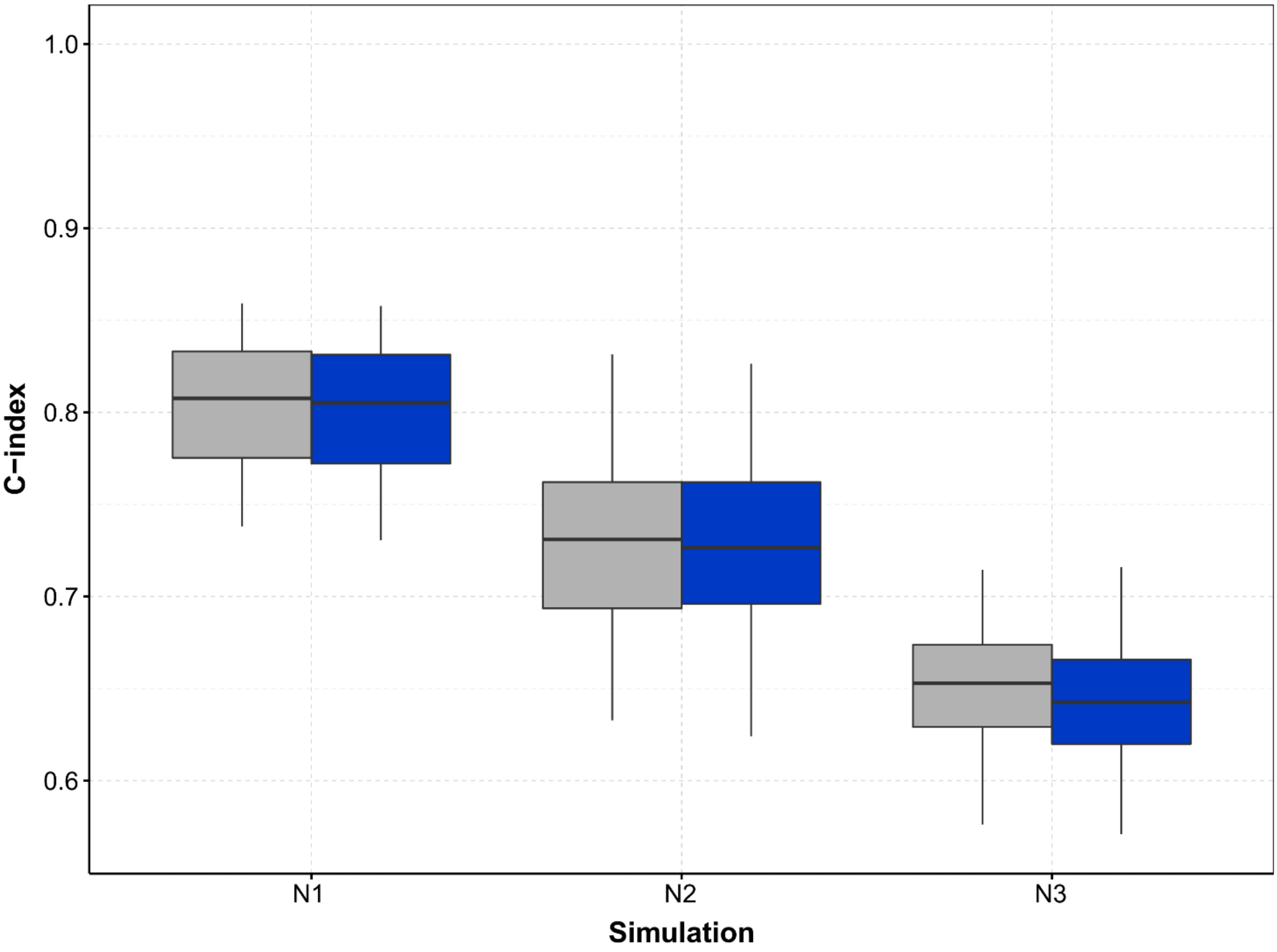

Supplementary Fig. S16. C-indices without pre-screening (gray boxplots) and with pre-screening in the optimal threshold (blue boxplots) for 3 simulated datasets. Equivalent of Figure 2C.

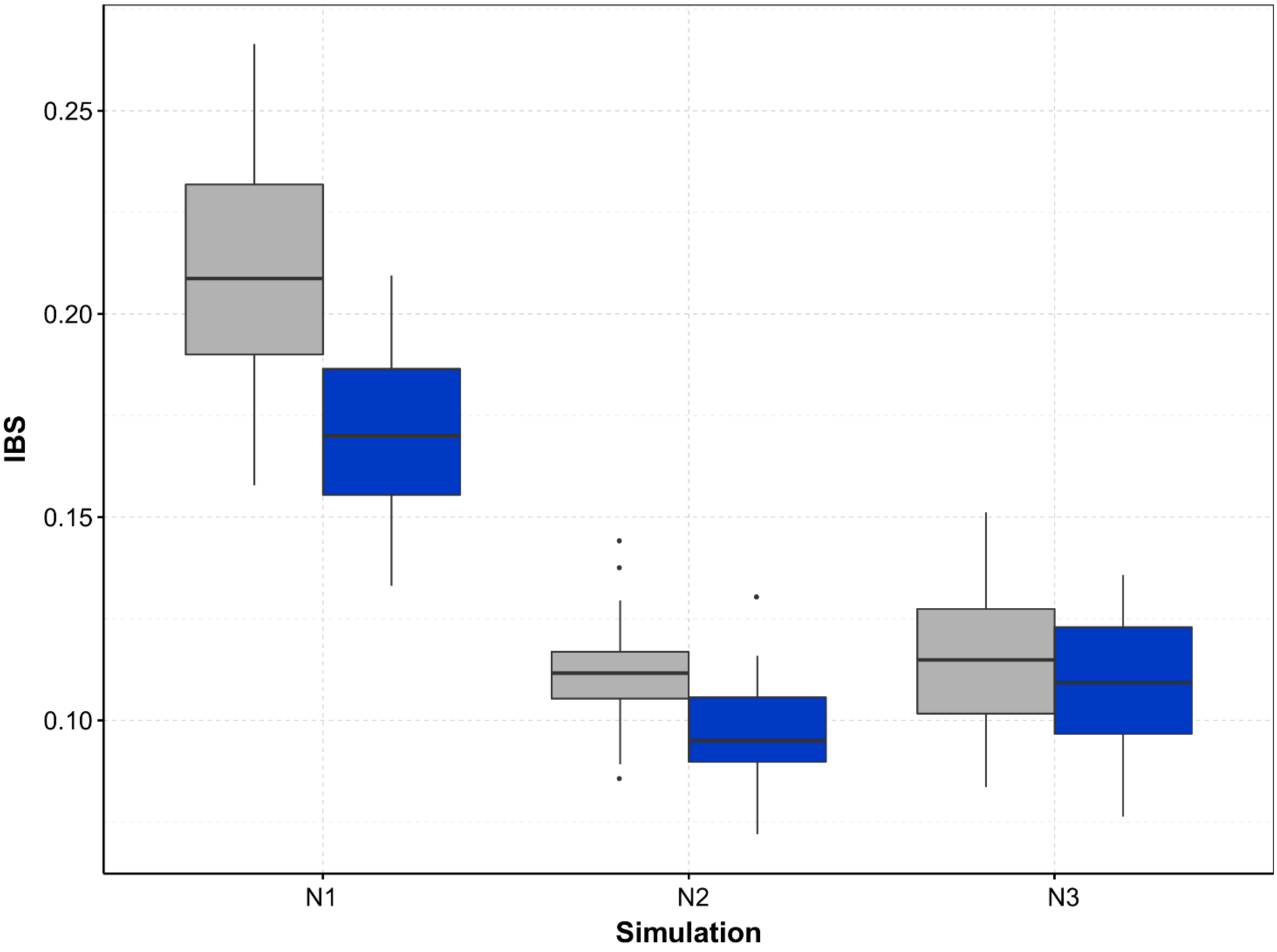

Supplementary Fig. S17. IBS without pre-screening (gray boxplots) and with pre-screening in the optimal threshold (blue boxplots) for 3 simulated datasets.

A

Cancer	ACC	KIRP	UVM	MESO	LGG	KIRC	CESC	PRAD
Correlation	0.993	0.995	0.994	0.996	0.998	0.995	0.998	0.996
Signif. Level	***	***	***	***	***	***	***	***
**Cancer**	LIHC	UCEC	BLCA	LAML	HNSC	BRCA	PAAD	LUAD
Correlation	0.998	0.998	0.999	0.998	0.999	0.999	0.997	0.999
Signif. Level	***	***	***	***	***	***	***	***

B

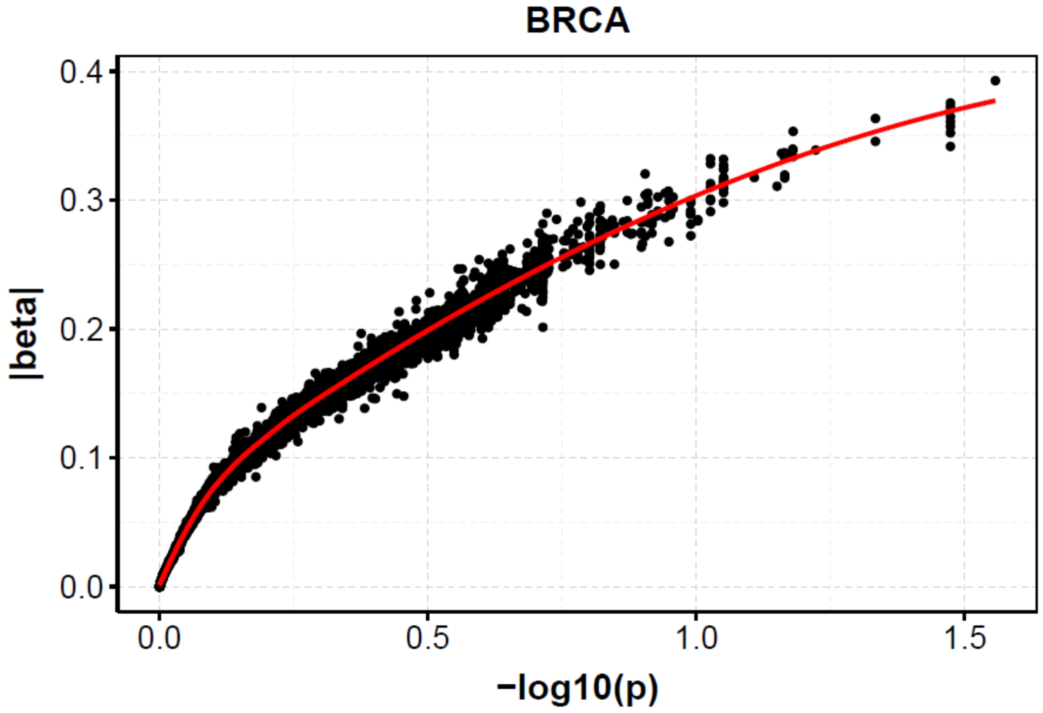

Supplementary Fig. S18. Spearman correlations between |beta| coefficients and p-values of univariate Cox models for each gene and significance level (A), and p-values as a function of |beta| for BRCA (B).

In graphics B, each dot represent a gene, and the red curve is loess regression over all points.

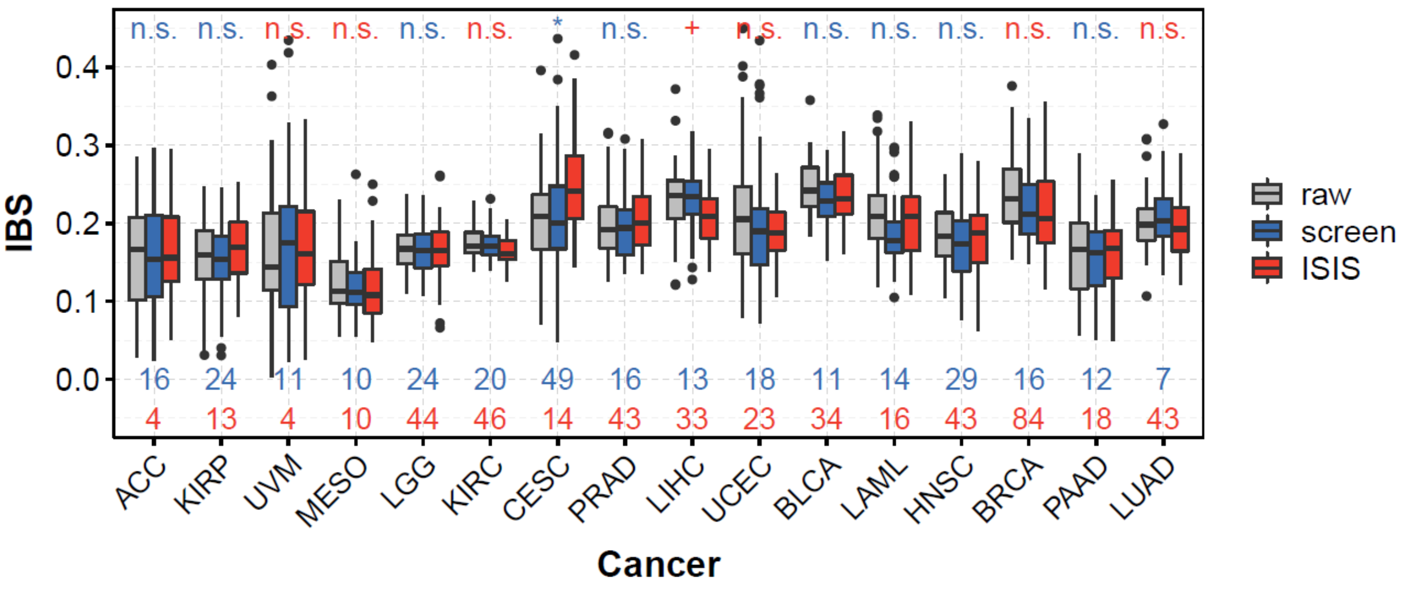

Supplementary Fig. S19. IBS obtained with the lasso without pre-screening (gray), with the lasso after the bi-dimensional pre-screening (blue), and with Independent Sure Independent Screening (ISIS, red).

Procedure detailed in Figure 1, that is we computed the IBS by 10 repetitions of a K-fold cross-validation (K=5). To test whether the median IBS obtained with ISIS and the bi-dimensional pre-screening are different, we computed p-values of a Wilcoxon test between screen and ISIS conditions (blue versus red boxplots, stars above the graphics). The 16 p-values are corrected with Benjamini-Hochberg method.

Red star: the median IBS is lower for ISIS.
Blue star: the median IBS is lower for the bi-dimensional pre-screening.

Blue numbers: number of genes selected by the lasso.
Red numbers: number of genes retained by ISIS.

***: $p \leq 0.001$, **: $p \leq 0.01$, *: $p \leq 0.05$, +: $p \leq 0.1$, n.s. : $p > 0.1$

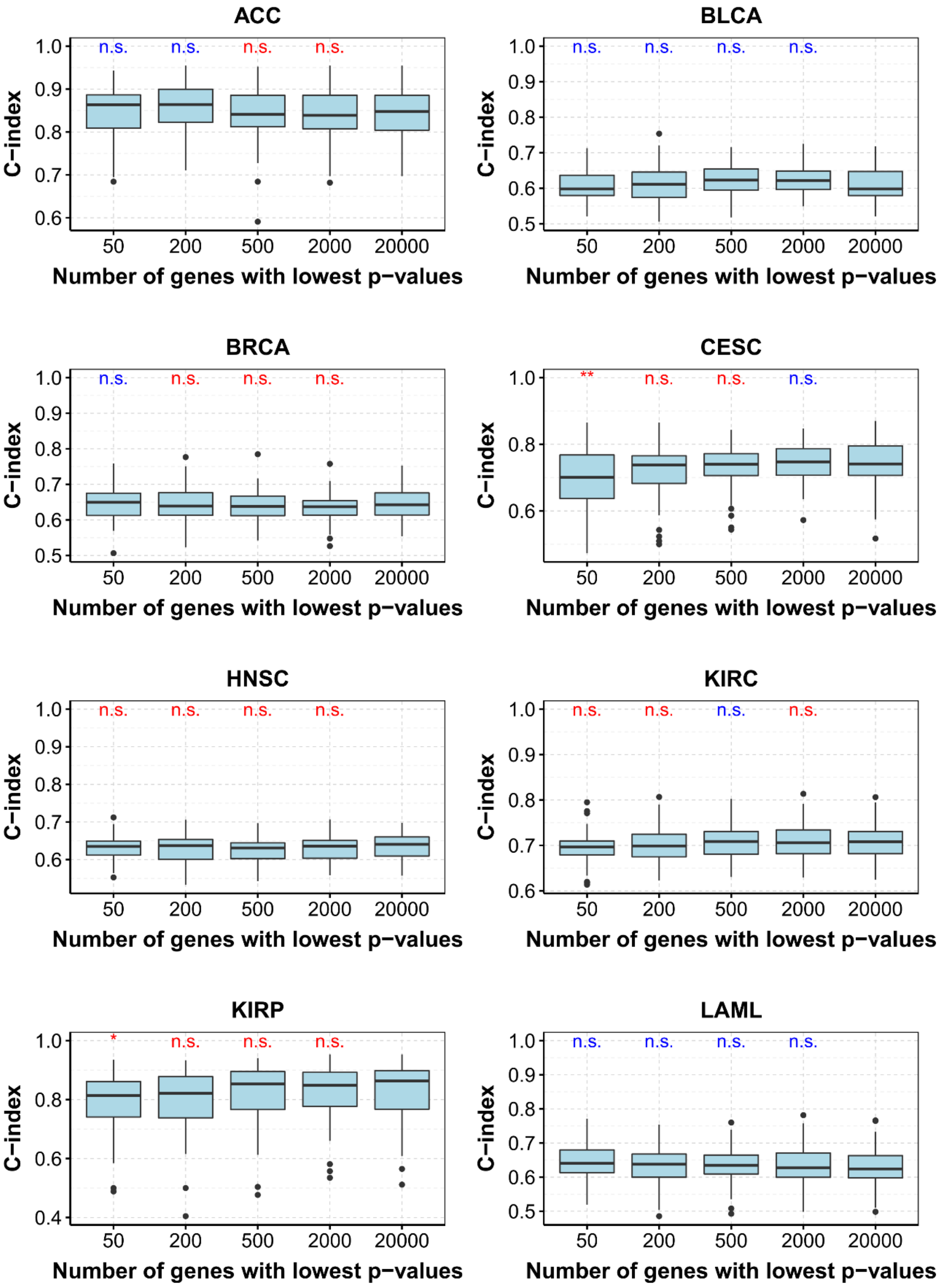

Supplementary Fig. S20. C-indices as a function of the number of genes with lowest univariate Cox model p-value used in the elastic net model.

Procedure detailed in Figure 1. Bilateral test. Blue (resp. red) indications correspond to improved (degraded) median performance.

***: $p \leq 0.001$, **: $p \leq 0.01$, *: $p \leq 0.05$, +: $p \leq 0.1$, n.s. : $p > 0.1$

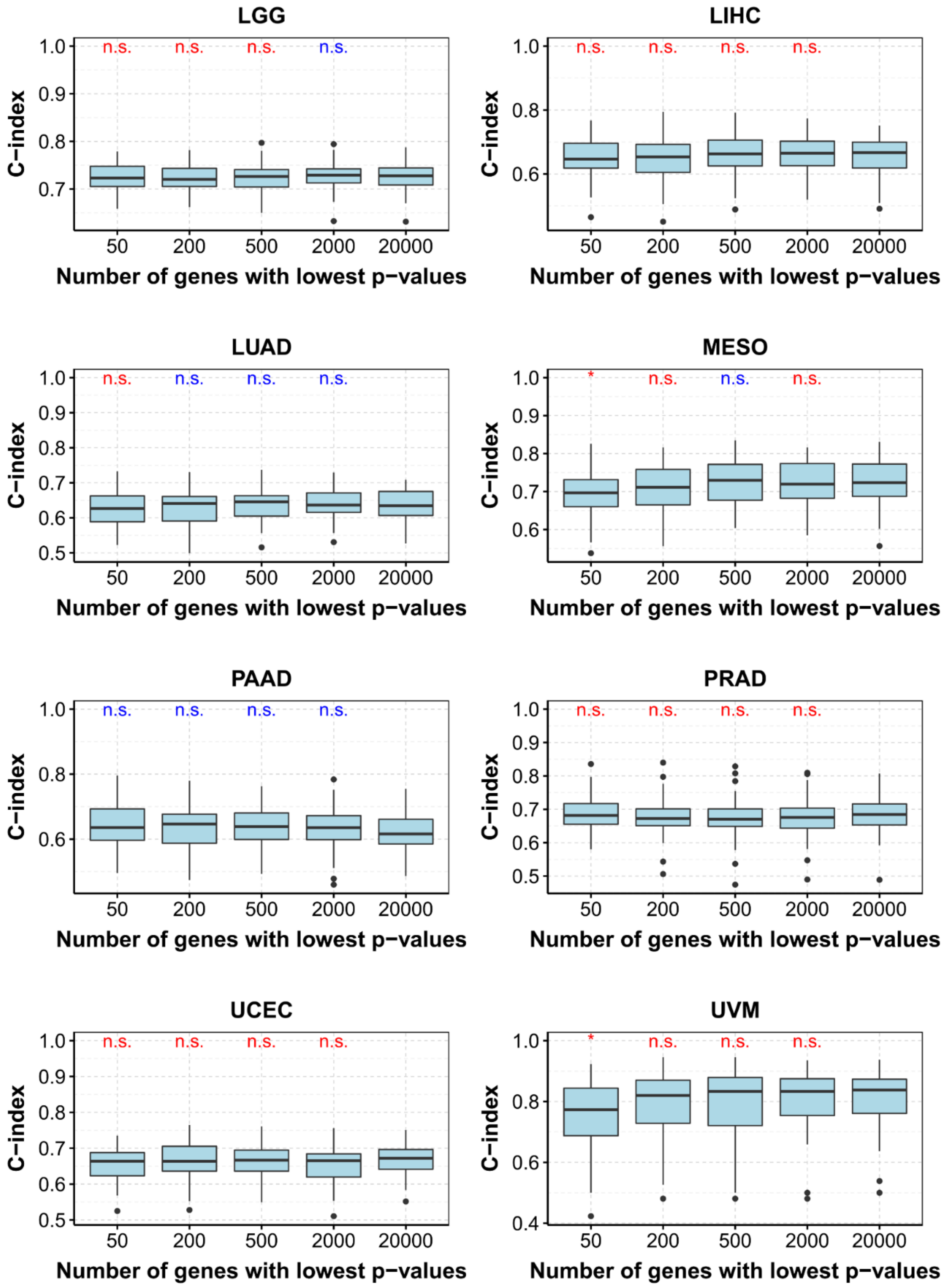

Supplementary Fig. S21. C-indices as a function of the number of genes with lowest univariate Cox model p-value used in the elastic net model.

Procedure detailed in Figure 1. Bilateral test. Blue (resp. red) indications correspond to improved (degraded) median performance.

***: $p \leq 0.001$, **: $p \leq 0.01$, *: $p \leq 0.05$, +: $p \leq 0.1$, n.s. : $p > 0.1$

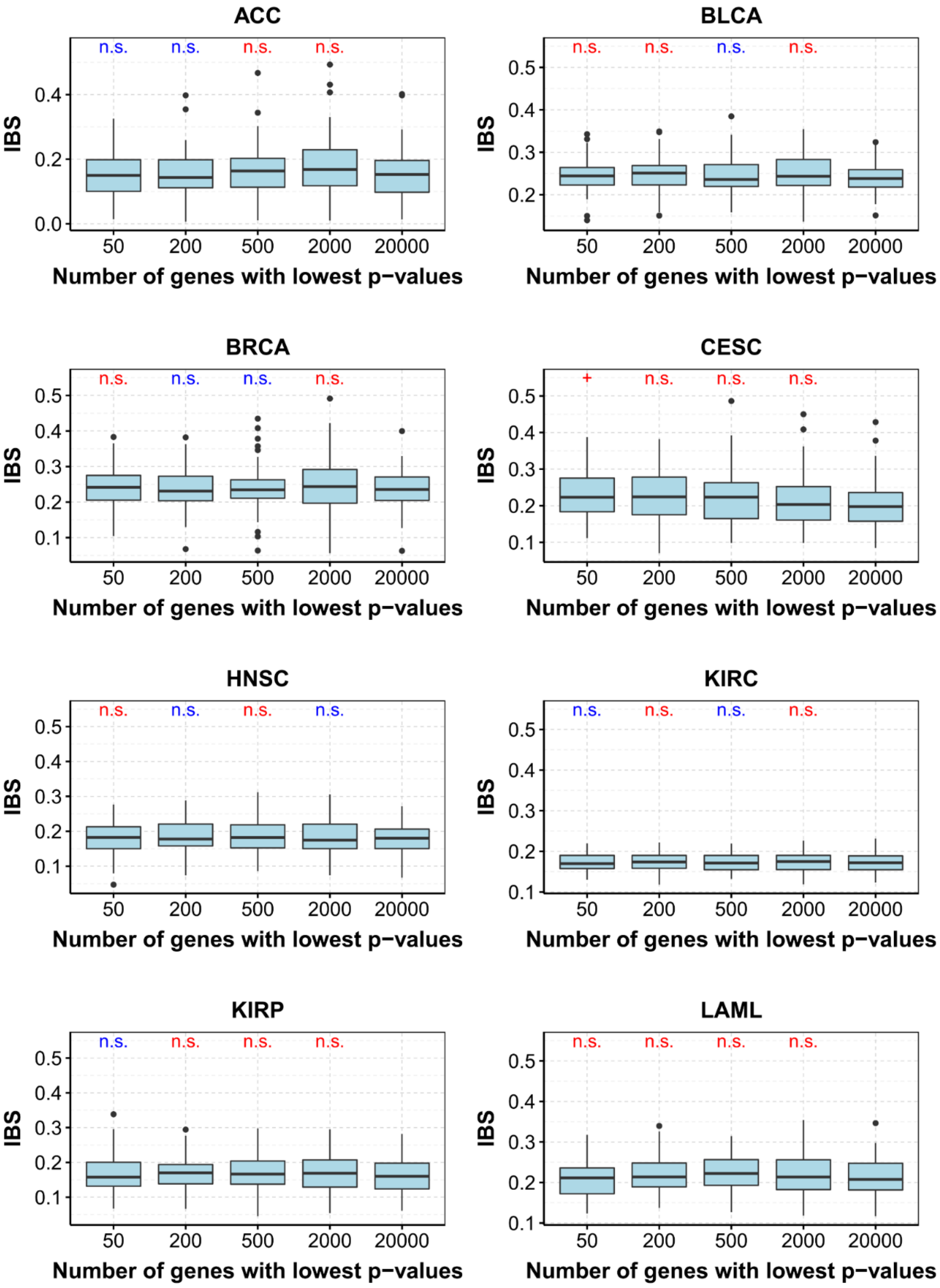

Supplementary Fig. S22. IBS as a function of the number of genes with lowest univariate Cox model p-value used in the elastic net model.

Procedure detailed in Figure 1. Bilateral test. Blue (resp. red) indications correspond to improved (degraded) median performance.

***: $p \leq 0.001$, **: $p \leq 0.01$, *: $p \leq 0.05$, +: $p \leq 0.1$, n.s. : $p > 0.1$

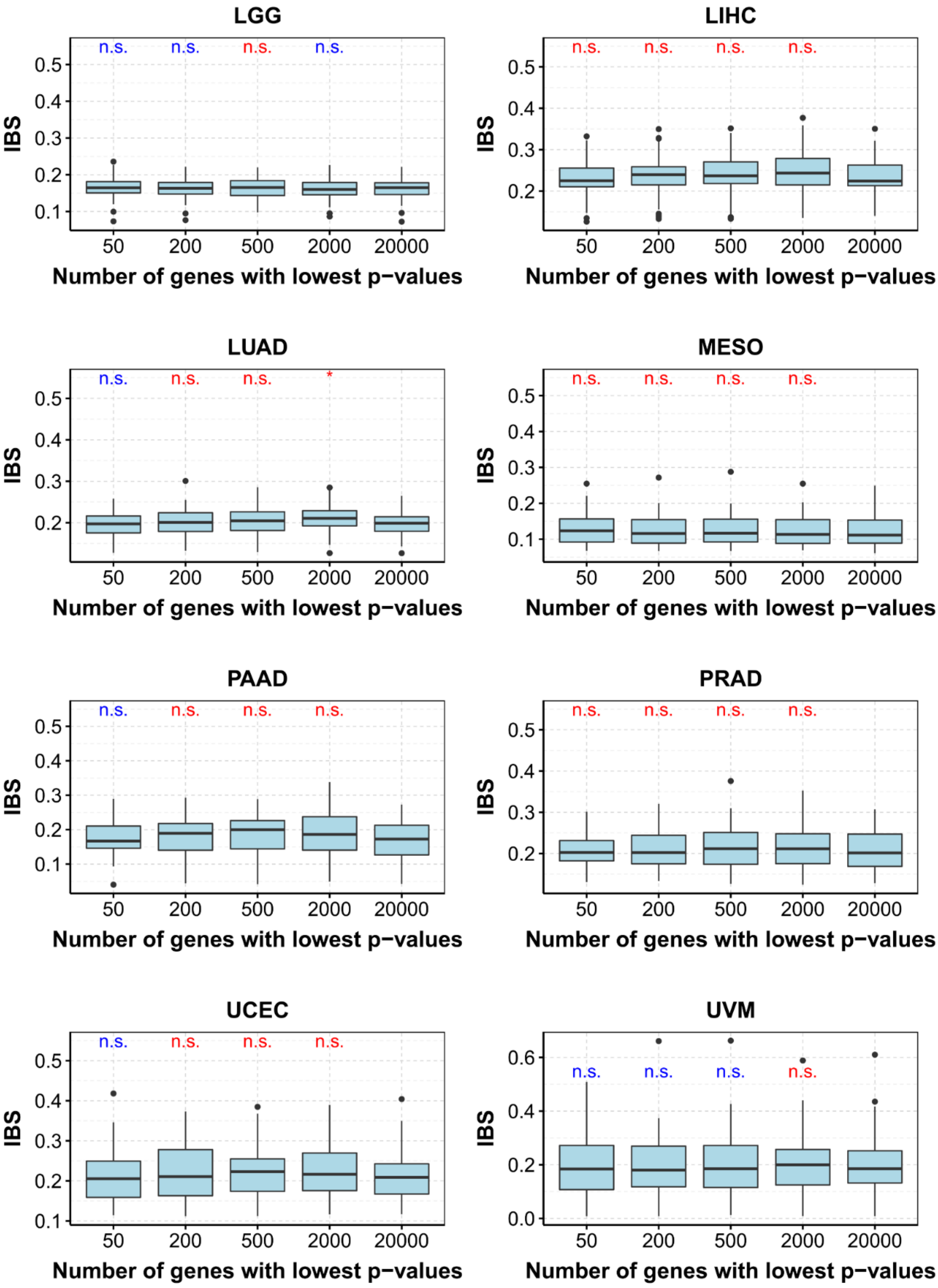

Supplementary Fig. S23. IBS as a function of the number of genes with lowest univariate Cox model p-value used in the elastic net model.

Procedure detailed in Figure 1. Bilateral test. Blue (resp. red) indications correspond to improved (degraded) median performance.

***: $p \leq 0.001$, **: $p \leq 0.01$, *: $p \leq 0.05$, +: $p \leq 0.1$, n.s. : $p > 0.1$
